# Effects of strain on boundary management: findings from a daily diary study and an experimental vignette study

**DOI:** 10.3389/fpsyg.2023.1149969

**Published:** 2023-10-24

**Authors:** Nicolas Mueller, Sophia Loeffelsend, Elke Vater, Regina Kempen

**Affiliations:** ^1^Department of Business Psychology, Aalen University of Applied Sciences, Aalen, Germany; ^2^Department of Work and Organizational Psychology, University of Osnabrück, Osnabrück, Germany; ^3^University of Würzburg, Würzburg, Germany

**Keywords:** boundary management, segmentation preference, integration enactment, strain, work-nonwork, daily diary study, experimental vignette study

## Abstract

**Introduction:**

Transformations in the work–nonwork interface highlight the importance of effectively managing the boundaries between life domains. However, do the ways individuals manage the boundaries between work and nonwork life change from one day to the next? If so, which antecedents may explain these intra-individual fluctuations in boundary management? Drawing on boundary management, spillover, and resource theories, we investigate daily changes in segmentation preferences and integration enactments as a function of experiencing strain in work and nonwork life. Assuming that changes in segmentation preferences reflect an individual’s strategy to regulate negative cross-role spillover, we suppose that strain increases individuals’ segmentation preferences; at the same time, however, it could force individuals to enact more integration.

**Methods:**

We test our assumptions with data from two studies with different methodological approaches. The first study uses a daily diary research design (Study 1, 425 participants with 3,238 daily observations) in which full-time professionals rated strain in work and nonwork life, segmentation preferences, and integration enactments every evening for 10 workdays. The second study uses an experimental vignette research design (Study 2, 181 participants), where we experimentally manipulated strain in work and nonwork life and investigated causal effects on participants’ hypothetical segmentation preferences.

**Results:**

The results of multilevel modeling analyses in Study 1 show that segmentation preferences and integration enactments fluctuate from day to day as a function of strain. More specifically, strain is related to preferring more segmentation but enacting more integration. Study 2 replicates the results of Study 1, showing that strain causally affects segmentation preferences.

**Discussion:**

This two-study paper is one of the first to address daily fluctuations in segmentation preferences and integration enactments, extending our knowledge of temporal dynamics in boundary management. Furthermore, it demonstrates that strain is an antecedent of these daily fluctuations, offering starting points for practical interventions.

## 1. Introduction

In the modern workplace, professionals face several challenges in managing the blurring of boundaries between their life domains (Olson-Buchanan and Boswell, 2006; Kniffin et al., 2021; Allen and French, 2023). Flexibilization and digitalization allow people to work anytime and anywhere, resulting in the progressive overlap of work and nonwork life (Allen et al., 2014; Rofcanin and Anand, 2020; Kniffin et al., 2021). As a result, people experience an increased cross-role spillover between work and nonwork life (Glavin and Schieman, 2012; Field and Chan, 2018; Cho et al., 2020), which highlights the significance of effectively managing the work–nonwork boundaries (Wayne et al., 2017; Wepfer et al., 2018; Ollier-Malaterre et al., 2019; Allen et al., 2021).

Most studies in the boundary management literature address inter-individual differences in the way people manage their work–nonwork boundaries and explore the effects of these inter-individual differences on the work–nonwork interface (Rothbard et al., 2005; Olson-Buchanan and Boswell, 2006; Powell and Greenhaus, 2010; Park et al., 2011; Kossek et al., 2012). For example, they show that people with weak work–nonwork boundaries experience more cross-role spillover than those with strong (for an overview, see Allen et al., 2014). In contrast, very few studies to date have considered the possibility that the way people manage their work–nonwork boundaries changes over time (Derks et al., 2016; van Steenbergen et al., 2018; Delanoeije et al., 2019; Hecht et al., 2022).

This neglect of possible changes is surprising because studying intra-individual fluctuations in preferred and actual work–nonwork boundaries and understanding the temporal dynamics of boundary management is crucial for boundary management theory and its practical application (Kreiner et al., 2009; Ammons, 2013; van Steenbergen et al., 2018). Consequently, previous studies call for more research that builds on dynamic models of work–nonwork experiences (Grzywacz and Marks, 2000; Allen and French, 2023) by using longitudinal and diary study designs, broadening our understanding of boundary management’s temporal nature (Capitano and Greenhaus, 2018; Wepfer et al., 2018; Smith et al., 2022).

The work–nonwork interface in terms of cross-role spillover is inherently dynamic (Ilies et al., 2007; Allen et al., 2019; Smith et al., 2022; Allen and French, 2023), and thus, boundary management may also change over time. However, it remains unclear how individuals manage their work–nonwork boundaries over short- (e.g., hours or days), mid- (e.g., weeks or months), and long-term (i.e., years or the entire lifespan) timeframes. In particular, research on daily changes in boundary management can be valuable given the volatile nature of day-to-day work–nonwork experiences (Allen and French, 2023). For example, it can be challenging to separate work and nonwork life in today’s connected and fast-paced world of work (Ollier-Malaterre et al., 2019; Allen et al., 2021), where disruptions of daily rhythms and routines have become the norm (Allen and French, 2023). Research on daily experiences and associated changes in boundary management can help us understand the micro-level of managing work–nonwork boundaries.

By examining intra-individual fluctuations in preferred and actual work–nonwork boundaries over time, researchers can gain insight into how individuals adapt their boundary management to changing internal or external factors (Grzywacz and Marks, 2000; van Steenbergen et al., 2018; Hecht et al., 2022). On the one hand, minor life events like daily hassles or uplifts in work and nonwork life may result in fluctuations (Kanner et al., 1981; Kempen et al., 2019). For example, individuals may wish to manage their boundaries differently on days when they experience more strain in work or nonwork life than on other days (Wepfer et al., 2018). On the other hand, major life events in nonwork life (e.g., the birth of a child) or in work life (e.g., changes in job responsibilities) could trigger lasting shifts in preferred and actual work–nonwork boundaries (Luhmann et al., 2012; Bakker et al., 2019; Allen et al., 2021; Smith et al., 2022). Regardless of whether life events are minor or major, they require individuals to adjust their work–nonwork boundaries to balance work and nonwork life (Allen et al., 2021).

In summary, the concept of boundary management is likely to vary over time based on several factors. Consequently, the current literature needs to pay more attention to these temporal processes (ten Brummelhuis and Bakker, 2012; Allen et al., 2019; Allen and French, 2023). Especially research on daily changes in boundary management can provide valuable insights into the dynamics and mechanisms by explaining *why* individuals’ boundary management may change from day to day (van Steenbergen et al., 2018). This knowledge could inform the development of interventions and policies that help individuals and organizations create more effective ways of managing work–nonwork boundaries.

Given these open research questions, the article’s overall objective is three-fold. First, we address intra-individual fluctuations in boundary management in terms of individuals’ preferred and enacted work–nonwork boundaries. Second, we investigate whether the daily experience of strain in work and nonwork life triggers such fluctuations in boundary management. Finally, given that these strain effects may vary across individuals as a function of their personal characteristics, we examine moderation effects by role involvement, which is considered a determinant of individuals’ boundary management (Capitano et al., 2017; Capitano and Greenhaus, 2018) and a moderator of day-to-day experiences in the work–nonwork interface (Williams and Alliger, 1994; Hecht et al., 2022).

The article makes several contributions to the boundary management literature: To our knowledge, this is the first article that investigates daily intra-individual fluctuations in both actual work–nonwork boundaries and individuals’ preferences (van Steenbergen et al., 2018). Thus, our article accommodates Allen and French’s (2023) recent call for conducting more research that explores change over time and deepens our understanding of day-to-day experiences in the work–nonwork interface. Moreover, we respond to the need for more research on the antecedents of boundary management variables by identifying work and nonwork strain as factors affecting actual work–nonwork boundaries and individuals’ preferences (Palm et al., 2020). Such strain effects could be crucial for practical applications, with significant implications for organizational measures that attempt to influence and improve employees’ boundary management and work–nonwork experiences. Finally, we address the research questions with data from a daily diary study and an experimental vignette study, providing data with high external and internal validity. Combining these two research designs allows us to draw solid conclusions.

## 2. Theoretical background and hypothesis development

### 2.1. Boundary management and spillover

Boundary management (Nippert-Eng, 1996; Ashforth et al., 2000; Clark, 2000) refers to how individuals manage work and nonwork life by creating, maintaining, and modifying boundaries around their life domains (for an overview, see Allen et al., 2014). Ashforth et al. (2000) and Clark (2000) conceptualize boundaries (or borders) as the limits or demarcation lines that separate different entities, such as work and nonwork life. The literature on boundary management states that individuals manage their work–nonwork boundaries in order to regulate the cross-role spillover between work and nonwork life (Edwards and Rothbard, 2000; Rothbard et al., 2021). Cross-role spillover refers to the transfer of characteristics and experiences from one life domain (e.g., work life) to another (e.g., nonwork life; Staines, 1980; Grzywacz and Marks, 2000). It can be negative (i.e., work–nonwork conflict; Greenhaus and Beutell, 1985; Eby et al., 2005) or positive (i.e., work–nonwork enrichment, enhancement, or facilitation; Edwards and Rothbard, 2000; Voydanoff, 2004; Greenhaus and Powell, 2006). Finally, positive and negative spillover can occur from work to nonwork life and nonwork to work life, resulting in four distinct work–nonwork experiences (Grzywacz and Marks, 2000).

Individuals can separate (i.e., segmentation) or blend (i.e., integration) their life domains (Nippert-Eng, 1996; Ashforth et al., 2000; Clark, 2000). Segmentation and integration represent two opposite poles on a continuum (Nippert-Eng, 1996). Concerning their consequences, the literature shows that integration promotes and segmentation reduces cross-role spillover (Allen et al., 2014). That is, strong boundaries prevent the spillover of thoughts, emotions, and activities from one life domain to another, and weak boundaries promote it (Edwards and Rothbard, 2000; Rothbard et al., 2021).

On the segmentation–integration continuum, individuals’ preferences (i.e., boundary management preference) and their actual behaviors (i.e., boundary management enactment; Ammons, 2013; Allen et al., 2021) can be distinguished. The boundary management preference is most frequently operationalized as a *segmentation preference* (Kreiner, 2006; Park et al., 2011; Derks et al., 2016; Althammer et al., 2021), and most researchers refer to the boundary management enactment as an *integration enactment* (van Steenbergen et al., 2018; Wepfer et al., 2018; Hirschi et al., 2022; Michel et al., 2022). Furthermore, researchers differentiate the direction of segmentation preferences and integration enactments (Allen et al., 2014; Michel et al., 2022). For example, some individuals could (prefer to) take phone calls from friends during working hours (i.e., *nonwork-to-work* direction) but would not (prefer to) answer work-related emails while spending time with their families (i.e., *work-to-nonwork* direction). Consequently, the present research considers work-to-nonwork and nonwork-to-work segmentation preferences and integration enactments.

### 2.2. Daily fluctuations in preferences and enactments

Most of the current literature positions boundary management as a temporally stable phenomenon, and many scholars view the way people manage their work–nonwork boundaries as unchanging (Hecht and Allen, 2009). However, the theories underlying boundary management research (boundary theory by Ashforth et al., 2000; work/family border theory by Clark, 2000) suggest that work–nonwork boundaries are dynamic and subject to the continuous influence of individuals and their environments (see also Ammons, 2013). Consequently, the first goal of this article is to address the daily fluctuations in segmentation preferences and integration enactments.

In Ashforth et al.’s (2000) boundary theory, boundaries are considered dynamic. They assume that boundaries are mainly influenced by “the psychological dynamics of daily role transitions” (p. 486). Moreover, boundaries are contextual, meaning that different contexts can shape them. Besides, boundary theory emphasizes that individuals actively and continuously work on their boundaries. Overall, Ashforth et al. (2000) assume that individuals’ role transitions and boundary work can change over time (see also Kreiner et al., 2009), which suggests examining boundary management at the daily micro-level.

Similarly, Clark (2000) views borders in her work/family border theory as dynamic. Her theory focuses on individuals as active border-crossers who make daily transitions between work and family life and shape their self-created borders continuously. Moreover, the theory states that boundary management is a collaborative process between more than one individual via negotiation and communication. Overall, boundary theory and work/family border theory consider boundary management a dynamic everyday phenomenon, supporting the notion that work–nonwork boundaries can change daily.

Besides these theoretical considerations, empirical research demonstrates temporal fluctuations in *integration enactments*. For example, van Steenbergen et al. (2018) investigated the effects of college students’ integration enactment on the conflict between home and school in a diary study over five consecutive days. They showed that 90% of the variance in integration enactment varied within participants. Delanoeije et al. (2019) conducted another diary study over 14 consecutive workdays. They demonstrated that 69% of the variance in employees’ work-to-home transitions and 55% of the variance in their home-to-work transitions was due to within-person variation. Finally, Hecht et al. (2022) found that within-person variance accounted for 59% and 44% of the overall variance in work and nonwork boundary permeation, respectively. Although these three articles operationalized integration enactments differentially, they provide the first evidence that integration enactments vary within individuals.

In contrast, we did not find any published study that investigated daily within-person fluctuations in *segmentation preferences*. Early research has operationalized segmentation preferences as personal traits that do not vary (e.g., Rothbard et al., 2005; Kreiner, 2006). Referring to these studies, the authors of the existing diary studies did not assess segmentation preference as a day-level variable (Derks et al., 2016; van Steenbergen et al., 2018; Delanoeije et al., 2019). For example, Derks et al. (2016) stated that “the [segmentation] preference itself is considered rather stable over time, and is not expected to fluctuate within the limited time frame of one week” (p. 1052). However, very little research has tested the assumption of temporal stability in segmentation preferences (Ammons, 2013), and some authors stated that the decision to consider segmentation preference as a stable person- versus fluctuating day-level variable could alter the results obtained (van Steenbergen et al., 2018; Delanoeije et al., 2019).

Taken together, researchers cannot rule out the possibility that individuals’ segmentation preferences change (Kreiner, 2006). Therefore, Ammons (2013) conducted interviews before and after a cultural change initiative in a large Fortune 500 company and demonstrated that some employees’ preferences changed. Whereas Ammons’s (2013) qualitative study addressed long-term changes (i.e., two measurements over 9 months), quantitative and diary studies have the potential to identify short-term fluctuations in segmentation preferences. Consequently, we conducted a daily diary study and considered segmentation preferences as a day-level variable.

In the case of our daily diary study, whole trait theory (Fleeson and Jayawickreme, 2015) provides the theoretical basis for the assumption of intra-individual fluctuations in segmentation preferences. Following this theory, traits can be highly variable due to variability in situations and experiences: If individuals experience different situations, their segmentation preferences can shift accordingly. Consequently, individuals who prefer to segment their life domains on average days may also wish to integrate on some special days (e.g., when their children are sick). In contrast, individuals who generally prefer to integrate could also wish to segment sometimes (e.g., when they are on vacation). Moreover, assuming that segmentation preferences can fluctuate reflects the idea that “most, if not all, psychological constructs include both a temporally stable, time-invariant, characteristic as well as a changing, or time-variant, characteristic” (Smith et al., 2022, p. 27). Consequently, we address within-person variations in not only integration enactments but also segmentation preferences.

*Hypothesis 1-1*^1^: Work-to-nonwork segmentation preference (a) and nonwork-to-work segmentation preference (b) fluctuate daily within individuals.

*Hypothesis 1-2*: Work-to-nonwork integration enactment (a) and nonwork-to-work integration enactment (b) fluctuate daily within individuals.

### 2.3. Effects of strain in work and nonwork life

The existing diary studies have addressed the consequences of daily changes in boundary management constructs (van Steenbergen et al., 2018; Delanoeije et al., 2019; Hecht et al., 2022). In contrast, more research is needed to investigate the factors that trigger these fluctuations (van Steenbergen et al., 2018). Consequently, the second goal of this article is to investigate possible antecedents of the fluctuations in segmentation preferences and integration enactments.

To identify possible antecedents, we refer to spillover theory (Staines, 1980; Grzywacz and Marks, 2000) that builds on an open-systems approach (Katz and Kahn, 1966), suggesting that work life affects nonwork life and vice versa. Grzywacz and Marks (2000) introduced a model of the work–nonwork interface that distinguishes positive and negative cross-role spillover. They propose that various work- and nonwork-related factors can influence the cross-role spillover. For example, they found that stressors in work and nonwork life, such as pressure and disagreements, were associated with more negative cross-role spillover. Specifically, work-related factors mainly contribute to the work-to-nonwork direction of spillover, whereas nonwork-related factors primarily affect the nonwork-to-work direction. Consequently, we decided to examine the strain people experience in work and nonwork life and its relationships to the ways they manage their work–nonwork boundaries.

Strain is a psychological, behavioral, and physiological response (Griffin and Clarke, 2011) that results from minor stressors (e.g., arguments or workload) and occurs on a daily basis (Brantley et al., 1988). Several studies demonstrate associations between boundary management variables and strain-related constructs such as stress, emotional exhaustion, and strain-based conflict between work and nonwork life (Voydanoff, 2005; Chen et al., 2009; Carlson et al., 2015; McNall et al., 2015; Bogaerts et al., 2018). These studies focus on the effects of boundary management variables on strain-related constructs. In contrast, we propose the reversed direction, making strain an interesting antecedent to investigate.

In general, we assume that strain is associated with preferring more segmentation. We build our prediction on Wepfer et al.’s (2018) suggestion that changes in boundary management could reflect a regulatory reaction to impaired well-being, strain, or a lack of recovery experiences: “Employees who feel exhausted and out of balance might start to segment both [life] domains to prevent a further decrease in well-being” (p. 737). We elaborate on this prediction using resource theories. First, conservation of resources theory (Hobfoll, 1989, 2001) proposes that individuals strive to build, maintain, and protect resources. Furthermore, people perceive a potential or actual loss of resources as threatening and try to prevent resource loss. Second, the work–home resources model (ten Brummelhuis and Bakker, 2012) links spillover and conservation of resources theory, suggesting that demands and resources exist in different life domains and contribute to positive and negative cross-role spillover, respectively. For example, demands in work life are associated with more negative work-to-nonwork spillover, and resources in work life are related to more positive work-to-nonwork spillover. The work–home resources model also addresses daily processes and predicts that temporal demands (e.g., the daily experience of strain in work and nonwork life) and volatile resources in life domains produce immediate effects in terms of negative and positive cross-role spillover, respectively.

Applying the spillover and resources theories’ propositions to the present research question, we suggest that experiencing strain in the life domain X threatens resources in the same life domain X (i.e., intra-domain risk of strain in X for resource loss in X) and, through the increased negative cross-role spillover, another life domain Y (i.e., inter-domain risk of strain in X for resource loss in Y). Preferring X-to-Y and Y-to-X segmentation could prevent resource loss in two ways. First, individuals who feel strained in X should prefer more X-to-Y segmentation to avoid resource loss in Y due to negative X-to-Y spillover (Ashforth et al., 2000; Allen et al., 2014; Wepfer et al., 2018). For example, individuals who value spending time with their families will strive to maintain this resource. However, high work demands and the associated risk of negative work-to-nonwork spillover threaten the resource, increasing their work-to-nonwork segmentation preference. Second, individuals who feel strained in X should prefer more Y-to-X segmentation to, for example, address the sources of strain in X and avoid cross-role interruptions by Y (Wepfer et al., 2018; Horvath et al., 2021; Perry et al., 2022). For example, individuals with high levels of work demands might turn off their private mobile phones to focus on their work tasks without being interrupted by personal phone calls. In sum, we assume that when individuals experience more strain in a life domain, they prefer more segmentation in both directions (i.e., work-to-nonwork and nonwork-to-work).

Individuals’ preferences are considered the most influential determinant of boundary management enactment (Matthews et al., 2010; Methot and LePine, 2016; Palm et al., 2020). Consequently, people should engage in more segmentation behaviors according to their preferences when they experience strain. However, we assume that enacting segmentation preferences is not always possible in both directions (i.e., work-to-nonwork versus nonwork-to-work) but depends on the life domain where people experience the strain (Ammons, 2013; Capitano and Greenhaus, 2018; Foucreault et al., 2018). First, individuals who feel strained in X should be able to enact more Y-to-X segmentation, which aligns with their stronger Y-to-X segmentation preference (Palm et al., 2020). For example, individuals preferring nonwork-to-work segmentation to avoid interruptions from nonwork life during stressful work periods should be able to enact this preference. This could manifest as being more productive during working hours and avoiding personal phone calls or messages during the workday. In contrast, individuals who feel strained in X might be unable to enact more X-to-Y segmentation (Kossek et al., 2012; Palm et al., 2020). For example, individuals facing a deadline to finish a project may have to take work home to complete their tasks in time. This could manifest as working late into the evening or answering emails during nonwork time.

The literature shows that work- and nonwork-related factors determine whether individuals can enact their preferences (Allen et al., 2014; Foucreault et al., 2018; Palm et al., 2020), leading to discrepancies between preferences and enactments (Ammons, 2013). For example, characteristics in work and nonwork life that cause the experience of strain (e.g., high levels of work or nonwork demands) could force individuals to integrate rather than segment their life domains regardless of their preferences. Such intention–behavior gaps (Sheeran and Webb, 2016) are likely to occur in stressful situations (Pfeffer et al., 2020). In sum, we assume that when individuals experience more strain in a life domain, they can enact their segmentation preference in one but not the other direction (i.e., work-to-nonwork versus nonwork-to-work).

*Hypothesis 2-1*: Strain in work life is positively related to an individual’s work-to-nonwork segmentation preference (a) and nonwork-to-work segmentation preference (b).

*Hypothesis 2-2*: Strain in work life is positively related to an individual’s work-to-nonwork integration enactment (a) and is negatively related to an individual’s nonwork-to-work integration enactment (b).

*Hypothesis 3-1*: Strain in nonwork life is positively related to an individual’s work-to-nonwork segmentation preference (a) and nonwork-to-work segmentation preference (b).

*Hypothesis 3-2*: Strain in nonwork life is negatively related to an individual’s work-to-nonwork integration enactment (a) and is positively related to an individual’s nonwork-to-work integration enactment (b).

### 2.4. The moderating role of work and nonwork role involvement

Grzywacz and Marks (2000) suggest that individual characteristics moderate the effects of work- and nonwork-related factors (e.g., strain in work and nonwork life) on the work–nonwork interface. Recently, Hecht et al. (2022) recommended examining stable personal characteristics, such as role saliences, as moderators of daily processes in boundary management. Consequently, the third goal of this article is to investigate work role involvement and nonwork role involvement as moderators of the strain effects on segmentation preferences and integration enactments.

On the basis of the person–situation interactionist perspective, suggesting that individuals’ thoughts and behaviors are the consequences of interactions between personal and situational factors (Lewin, 1935; Buss, 1977), we suppose that fluctuations in segmentation preferences and integration enactments result from interactions between strain in work and nonwork life (i.e., situational factors) and work and nonwork role involvement (i.e., personal factors). That is, we suggest that individuals who are more involved in a life domain adjust their boundary management differently to experiencing strain compared to individuals who are less involved.

Previous between-person research has addressed relationships between boundary management variables and role involvement (Olson-Buchanan and Boswell, 2006; Boswell and Olson-Buchanan, 2007; Reinke and Gerlach, 2022) or related constructs (e.g., role identity salience; Capitano and Greenhaus, 2018). Role involvement represents the degree to which individuals’ roles are central to their self-concepts (Kanungo, 1982). Capitano et al. (2017) demonstrate that individuals who strongly identify with one role are motivated to enact and integrate that role into other roles (i.e., enactment effect) and to protect and segment it from other roles (i.e., protection effect). Consequently, individuals with a high work role involvement prefer and enact work-to-nonwork integration and nonwork-to-work segmentation, and individuals with a high nonwork role involvement prefer and enact nonwork-to-work integration and work-to-nonwork segmentation. Identity theories provide the basis for explaining these relationships, suggesting that individuals’ identification with their roles shapes their intentions and behaviors in identity-consistent ways (for an overview, see Allen and French, 2023). Altogether, work and nonwork role involvement affect segmentation preferences and integration enactments. However, these effects occur at the between-person level. In contrast, we are interested in within-person fluctuations, focusing on the interaction rather than the main effects of work and nonwork role involvement (Hecht et al., 2022).^2^

First, individuals more involved in the life domain X are more motivated to integrate X into Y than those less involved in X (Capitano et al., 2017; Capitano and Greenhaus, 2018). This should be true even more in case of strain in X. More specifically, experiencing strain in X may tempt highly involved individuals to integrate X into Y, representing identity-consistent intentions and behaviors (Allen and French, 2023). For example, individuals highly involved in work life should be more likely to bring work tasks home with them in times of high work demands than less involved individuals. Consequently, we predict that when individuals experience strain in X, those who are more versus less involved in X will be less likely to prefer more X-to-Y segmentation (i.e., weaker strain effect) and more likely to enact more X-to-Y integration (i.e., stronger strain effect).

Second, changing their X-to-Y integration tendency towards more segmentation as a response to experiencing strain in Y would mean distancing themselves from an integral part of their self-concept (Kanungo, 1982; Thoits, 1992; Stryker and Burke, 2000). As a result, individuals more involved in X should not prefer and enact more X-to-Y segmentation following strain in Y compared to individuals less involved in X (Capitano et al., 2017; Capitano and Greenhaus, 2018). For example, individuals highly involved in work life should be less affected by strain in nonwork life in terms of changes in their work-to-nonwork preference and enactment than less involved individuals. Consequently, we predict that when individuals experience strain in Y, those who are more versus less involved in X will be less likely to prefer more X-to-Y segmentation (i.e., weaker strain effect) and less likely to enact less X-to-Y integration (i.e., weaker strain effect).

Third, individuals more involved in X should be specifically prone to build, maintain, and protect resources in X (Hobfoll, 1989, 2001; ten Brummelhuis and Bakker, 2012). When they feel strained in X, they might try to cope with its strain-causing factors without being interrupted by Y-related issues (Horvath et al., 2021; Perry et al., 2022). Consequently, they should focus on X and separate X from Y to, for example, avoid distraction that can result from negative Y-to-X spillover (e.g., cross-role interruptions; Capitano and Greenhaus, 2018). For example, individuals highly involved in work life may be more likely to focus on their work tasks when they are strained than less involved individuals in order to avoid distractions from nonwork life. Consequently, we predict that when individuals experience strain in X, those who are more versus less involved in X will be more likely to prefer more Y-to-X segmentation (i.e., stronger strain effect) and more likely to enact less Y-to-X integration (i.e., stronger strain effect).

Finally, individuals more involved in X may use specific strategies to regulate negative Y-to-X spillover (Wepfer et al., 2018) and to avoid resource loss in X (Hobfoll, 1989, 2001; ten Brummelhuis and Bakker, 2012). In other words, individuals more involved in X might be more motivated to protect resources in X and prevent resource loss than those less involved in X (Capitano et al., 2017; Capitano and Greenhaus, 2018). As resource loss in X can result from negative Y-to-X spillover, they might prefer and enact Y-to-X segmentation even more in case of strain in Y. For example, individuals highly involved in work life should be more motivated to protect their work-related resources and likely to perceive strain in nonwork life and the related risk of negative nonwork-to-work spillover as more threatening than less involved individuals. Consequently, we predict that when individuals experience strain in Y, those who are more versus less involved in X will be more likely to prefer more Y-to-X segmentation (i.e., stronger strain effect) and less likely to enact more Y-to-X integration (i.e., weaker strain effect).

*Hypothesis 4-1*: Work role involvement moderates the relationships between strain in work and nonwork life and work-to-nonwork segmentation preference (a) and nonwork-to-work segmentation preference (b): The higher the work role involvement, the weaker the positive relationship between strain in work life and work-to-nonwork segmentation preference, the weaker the positive relationship between strain in nonwork life and work-to-nonwork segmentation preference, the stronger the positive relationship between strain in work life and nonwork-to-work segmentation preference, and the stronger the positive relationship between strain in nonwork life and nonwork-to-work segmentation preference.

*Hypothesis 4-2*: Work role involvement moderates the relationships between strain in work and nonwork life and work-to-nonwork integration enactment (a) and nonwork-to-work integration enactment (b): The higher the work role involvement, the stronger the positive relationship between strain in work life and work-to-nonwork integration enactment, the weaker the negative relationship between strain in nonwork life and work-to-nonwork integration enactment, the stronger the negative relationship between strain in work life and nonwork-to-work integration enactment, and the weaker the positive relationship between strain in nonwork life and nonwork-to-work integration enactment.

*Hypothesis 5-1*: Nonwork role involvement moderates the relationships between strain in work and nonwork life and work-to-nonwork segmentation preference (a) and nonwork-to-work segmentation preference (b): The higher the nonwork role involvement, the stronger the positive relationship between strain in work life and work-to-nonwork segmentation preference, the stronger the positive relationship between strain in nonwork life and work-to-nonwork segmentation preference, the weaker the positive relationship between strain in work life and nonwork-to-work segmentation preference, and the weaker the positive relationship between strain in nonwork life and nonwork-to-work segmentation preference.

*Hypothesis 5-2*: Nonwork role involvement moderates the relationships between strain in work and nonwork life and work-to-nonwork integration enactment (a) and nonwork-to-work integration enactment (b): The higher the nonwork role involvement, the weaker the positive relationship between strain in work life and work-to-nonwork integration enactment, the stronger the negative relationship between strain in nonwork life and work-to-nonwork integration enactment, the weaker the negative relationship between strain in work life and nonwork-to-work integration enactment, and the stronger the positive relationship between strain in nonwork life and nonwork-to-work integration enactment.

## 3. Study 1: daily diary study

In Study 1, we investigated daily fluctuations in segmentation preferences and integration enactments as a function of strain in work and nonwork life. We used a daily diary study with a screening survey and 10 daily questionnaires.

### 3.1. Materials and methods of Study 1

#### 3.1.1. Open science, ethical review, and funding

We pre-registered the daily diary study at PsychArchives (Mueller et al., 2022b). The pre-registration was peer-reviewed by anonymous reviewers via PsychLab,^3^ a service of the Leibniz Institute for Psychology (ZPID), which funded data collection afterward. The study was reviewed and approved by the Ethics Committee of the University of Osnabrück. The participants provided their written informed consent to participate in this study.

#### 3.1.2. Participants and procedure

We recruited participants through the online panel provider Bilendi & respondi.^4^ In the first step, a screening survey informed participants about the content and procedure of the study, compensation, data protection, voluntariness, and anonymity; and they gave informed consent. Following van Steenbergen et al. (2018), we defined the terms “work life” and “nonwork life” at the beginning of each questionnaire by explaining that “nonwork life” represents the private life, including family, friends, partners, and hobbies, and “work life” refers to the professional life associated with their full-time jobs (e.g., employment). Next, sociodemographic variables and work and nonwork role involvement were measured. The screening survey selected participants based on the following inclusion criteria: German-speaking, aged between 18 and 67 years, working full-time (i.e., for at least 35 h per week), working each day from Monday to Friday, and having no planned vacation within the study period. Additionally, we excluded data from participants who did not pass an attention check (“Please select ‘neither nor’ for this item”) or completed the screening survey exceptionally fast (i.e., a minimum response time of fewer than 60 s; Huang et al., 2012; Porter et al., 2019). In sum, the online panel provider recruited 500 participants who met all inclusion criteria and agreed to participate in the study.

The qualified participants completed daily questionnaires scheduled on 10 consecutive workdays from Monday to Friday in the 2 weeks following the recruiting and screening process. In the daily questionnaires, we asked participants to complete the measures of strain in work and nonwork life, work-to-nonwork and nonwork-to-work segmentation preferences, and work-to-nonwork and nonwork-to-work integration enactments. The questionnaires were sent to the participants at the end of each workday and could be filled out between 6 and 12 p.m. We did not send daily questionnaires on weekends. The daily questionnaires were kept as short as possible (Ohly et al., 2010) and could be completed in 2 min. Participation in the daily questionnaires was only possible if the participants indicated they had worked that day. As an incentive for participating in the study and completing as many daily questionnaires as possible, participants received points for each completed questionnaire, which could be converted into a voucher or donated. The online panel provider administered the compensation for participation in the study.

In total, the participants filled out 3,309 daily questionnaires; however, 46 daily questionnaires were excluded due to short response times (i.e., a minimum response time of fewer than 45 s). Of the 500 invited participants, 52 did not participate in any daily questionnaire, and 23 participated only once. These participants were excluded from the analyses (Nezlek, 2012). Consequently, the final sample consisted of *N* = 425 participants and 3,238 daily questionnaires with, on average, 7.6 daily questionnaires per participant.

Of the 425 participants included in the analyses, 46.6% were women, and the mean age was 43.50 years (*SD* = 13.00, range: 18–67). About 72.5% indicated living in a partnership, 23.8% cared for at least one child under 18 years who lived in the same household, and 7.8% cared for other people in private life (e.g., elderly parents). Approximately one-third of the participants (32.7%) had a university degree, 28.2% had a high school diploma or a university of applied sciences entrance qualification, 32.2% had a secondary school leaving certificate, and 6.8% had a secondary modern school qualification. On average, participants worked 40.79 h per week (*SD* = 4.32, range: 35–70) and had worked for their current employer for 12.29 years (*SD* = 10.99, range: 0–50). Some participants indicated they were self-employed (7.8%), and 30.8% had supervisory roles. On average, participants teleworked 1.67 days per week (*SD* = 2.08, range: 0–7).

Difference tests between excluded participants (i.e., those who completed less than two daily questionnaires) and included participants revealed no significant differences in the variables assessed in the screening survey (see Supplementary Table S1).

#### 3.1.3. Measures

All items were in German. Scales developed in English were translated and back-translated before (Brislin, 1980).

##### 3.1.3.1. Strain

We measured strain in work and nonwork life with self-constructed scales adapted from De Gieter et al. (2018), Littman et al. (2006), and Motowidlo et al. (1986). The strain measures comprised three items (Shrout and Lane, 2012), which we selected and adapted from several single- and multiple-item measures. These items assessed strain based on participants’ subjective perceptions (Houdmont et al., 2019). More specifically, participants were asked to think about their experiences during the day and rate the items by adjusting a slider on a scale between 0 and 100. Example items were “Please rate the amount of strain you experienced because of your work today” (strain in work life) and “Please rate the amount of strain you experienced because of your nonwork life today” (strain in nonwork life). The endpoint labels varied across the items (see the pre-registration; Mueller et al., 2022b). Visual analog scales were chosen because Likert scales might be too coarse to detect small daily fluctuations in strain. Cronbach’s alpha ranged from 0.95 to 0.98, with an average alpha of 0.97, for the measurement waves of strain in work life, and from 0.96 to 0.99, with an average alpha of 0.98, for the measurement waves of strain in nonwork life.

##### 3.1.3.2. Segmentation preference

Work-to-nonwork and nonwork-to-work segmentation preferences were measured with Michel et al.’s (2022) Work Segmentation Preference and Nonwork Segmentation Preference scales, which they adapted from Kreiner (2006). Both subscales were assessed with four items, which we adapted to reflect participants’ day-level preferences (van Steenbergen et al., 2018; Delanoeije et al., 2019). Example items were “Today, I did not like to think about my work life outside my working hours” (work-to-nonwork segmentation preference) and “Today, I did not like to think about my nonwork life while I was at work” (nonwork-to-work segmentation preference). Participants rated all items on a Likert scale from 1 (*strongly disagree*) to 7 (*strongly agree*). Cronbach’s alpha ranged from 0.91 to 0.97, with an average alpha of 0.96, for the measurement waves of work-to-nonwork segmentation preference, and from 0.94 to 0.97, with an average alpha of 0.96, for the measurement waves of nonwork-to-work segmentation preference.

##### 3.1.3.3. Integration enactment

Work-to-nonwork and nonwork-to-work integration enactments were measured with Desrochers et al.’s (2005) Work-Family Integration-Blurring Scale (see also van Steenbergen et al., 2018, as an example for using this scale in a daily diary study). Both subscales were measured with three items, which we adapted to reflect participants’ day-level enactments. Example items were “Today, I tended to integrate my work life into my nonwork duties” (work-to-nonwork integration enactment) and “Today, I tended to integrate my nonwork life into my work duties” (nonwork-to-work integration enactment). Participants rated all items on a Likert scale from 1 (*strongly disagree*) to 7 (*strongly agree*). Cronbach’s alpha ranged from 0.84 to 0.93, with an average alpha of 0.89, for the measurement waves of work-to-nonwork integration enactment, and from 0.84 to 0.93, with an average alpha of 0.89, for the measurement waves of nonwork-to-work integration enactment.

##### 3.1.3.4. Role involvement

We used Frone and Rice’s (1987) Job Involvement Scale and Family Involvement Scale, which they adapted from Kanungo (1982), to assess work and nonwork role involvement. Both subscales were measured with four items, which we adapted to the present study context (e.g., rephrasing the words “job” and “family” to “work” and “nonwork”). Example items were “I am very much involved in my work role” (work role involvement) and “I am very much involved in my nonwork role” (nonwork role involvement). Participants rated all items on a Likert scale from 1 (*strongly disagree*) to 7 (*strongly agree*). Cronbach’s alpha was 0.84 for work role involvement and 0.88 for nonwork role involvement.

##### 3.1.3.5. Control variables

The literature suggests that several sociodemographic variables may covary with boundary management variables (e.g., Bulger et al., 2007; Palm et al., 2020). Consequently, we measured the following variables to assess their possible influence on segmentation preferences and integration enactments: gender, age, education level, living in a partnership, care for children or other people in private life (e.g., elderly parents), working hours per week, self-employment, supervisory role, organizational tenure, work-related availability in nonwork time (Likert scale from 1 = *never* to 5 = *very often*), teleworking days per week, and the impact of the COVID-19 pandemic on boundary management (Likert scale from 1 = *no impact* to 5 = *extremely strong impact*). We planned to control for those sociodemographic variables significantly associated with the boundary management variables (see Mueller et al., 2022b).

#### 3.1.4. Analytic strategy

All analyses were performed using R (version 4.2.0; R Core Team, 2022). We analyzed the hierarchically structured (i.e., daily observations nested within individuals) data with a two-level multilevel modeling approach (Nezlek, 2020) using the lme4 package (Bates et al., 2015; optimizer bobyqa). Multilevel models tested lower-level direct effects (i.e., strain in work and nonwork life modeled as Level-1 predictors), cross-level direct effects (i.e., work and nonwork role involvement modeled as Level-2 predictors), and cross-level interaction effects on work-to-nonwork and nonwork-to-work segmentation preferences and integration enactments. The multilevel model-building process involved a sequence including five steps (Aguinis et al., 2013).

First, a null model without any predictors, including only the randomly varying intercepts, provided information about how the total variances of the segmentation preferences and integration enactments were distributed across the two levels of the model (e.g., the intraclass correlation coefficient, ICC). Second, a control model included control variables. Third, a random intercept and fixed slope model included Level-1 and Level-2 predictors. Fourth, a random intercept and slope model allowed the slopes of the Level-1 predictors to vary randomly across Level-2 units. Finally, a cross-level interaction model included interaction terms and tested whether the Level-1 relationships varied as a function of Level-2 variables.

Before conducting the analyses, Level-1 predictors were person-mean centered. Following Nezlek (2012), continuous Level-2 predictors were entered as grand-mean centered, and categorical Level-2 predictors were entered as uncentered. Multilevel models were estimated using maximum likelihood (ML) estimation. To compare nested models, we used likelihood ratio tests of model deviances. Marginal and conditional *R*^2^ values (i.e., “pseudo” *R*^2^ values) were calculated following Nakagawa et al. (2017).

### 3.2. Results of Study 1

Table 1 reports descriptive statistics, reliabilities, and correlations (aggregated to the person and day level) for all variables. Sociodemographic variables were used as control variables in the multilevel models when they significantly correlated with the criteria at the person level.

**Table 1:** Descriptive statistics, reliabilities, and correlations for all variables [Study 1].

Variable	*M*	*SD*	1	2	3	4	5	6	7	8	9
1. Strain work	34.23	23.00	(0.97)	0.39^***^	0.04	0.02	0.38^***^	0.18^***^	0.13^**^	−0.09	−0.20^***^
2. Strain nonwork	18.72	18.47	0.54^***^	(0.98)	−0.13^***^	−0.02	0.28^***^	0.42^***^	0.09	−0.05	−0.27^***^
3. Seg. pref. w-to-n	5.52	1.19	0.05	−0.15^**^	(0.96)	0.37^***^	−0.34^***^	−0.26^***^	−0.22^***^	0.32^***^	0.08^***^
4. Seg. pref. n-to-w	4.46	1.30	0.02	−0.03	0.44^***^	(0.96)	−0.16^***^	−0.31^***^	0.13^**^	−0.09	0.15^***^
5. Int. enac. w-to-n	2.85	1.24	0.45^***^	0.38^***^	−0.42^***^	−0.20^***^	(0.89)	0.55^***^	0.32^***^	−0.25^***^	−0.18^***^
6. Int. enac. n-to-w	3.04	1.18	0.28^***^	0.51^***^	−0.34^***^	−0.39^***^	0.66^***^	(0.89)	0.15^**^	−0.11^*^	−0.18^***^
7. Work role invol.	3.96	1.15	0.12^***^	0.07^**^	−0.19^***^	0.08^***^	0.25^***^	0.12^***^	(0.84)		
8. Nonwork role invol.	5.33	1.09	−0.08^***^	−0.05	0.26^***^	−0.06^*^	−0.20^***^	−0.09^***^	−0.49^***^	(0.88)	
9. Age	43.50	13.00	−0.23^***^	−0.34^***^	0.10^*^	0.18^***^	−0.24^***^	−0.26^***^	0.02	−0.11^*^	—
10. Working hours	40.79	4.32	0.13^**^	0.02	−0.05	−0.07	0.18^***^	0.06	0.16^***^	−0.14^**^	0.04
11. Gender^a^	—	—	−0.13^**^	−0.07	−0.03	0.01	−0.08	−0.03	−0.06	0.00	0.21^***^
12. Education level^b^	—	—	0.07	0.07	−0.13^**^	−0.04	0.16^**^	0.10^*^	0.11^*^	−0.03	−0.15^**^
13. Partnership^c^	—	—	0.07	0.03	−0.04	−0.11^*^	0.01	0.03	−0.02	0.15^**^	0.01
14. Care for children^c^	—	—	0.07	0.14^**^	−0.09	−0.04	0.06	0.02	0.04	0.09	−0.10^*^
15. Care for parents^c^	—	—	0.02	0.03	−0.02	−0.02	0.00	0.05	0.07	0.00	0.17^***^
16. Self-employment^c^	—	—	−0.12^*^	−0.12^*^	−0.14^**^	0.01	0.07	0.03	0.16^***^	−0.11^*^	0.20^***^
17. Supervisory role^c^	—	—	0.02	−0.04	−0.05	0.07	0.07	0.00	0.22^***^	−0.10^*^	0.13^**^
18. Org. tenure	12.29	10.99	−0.12^*^	−0.18^***^	0.09	0.15^**^	−0.19^***^	−0.22^***^	−0.03	0.01	0.59^***^
19. Availability	2.59	0.99	0.23^***^	0.14^**^	−0.16^***^	−0.03	0.37^***^	0.18^***^	0.39^***^	−0.13^**^	−0.12^*^
20. Telework	1.67	2.08	−0.05	0.00	−0.03	0.04	0.08	0.02	0.05	−0.08	0.03
21. COVID-19 impact	2.61	1.04	0.24^***^	0.23^***^	−0.04	−0.01	0.23^***^	0.19^***^	0.11^*^	0.07	−0.08

Variable	10	11	12	13	14	15	16	17	18	19	20	21
1. Strain work	0.10^***^	−0.10^***^	0.07^**^	0.06^*^	0.05	−0.01	−0.08^***^	0.00	−0.11^***^	0.19^***^	−0.02	0.20^***^
2. Strain nonwork	0.03	−0.04	0.07^**^	0.05	0.11^***^	0.00	−0.08^***^	−0.02	−0.14^***^	0.12^***^	−0.01	0.19^***^
3. Seg. pref. w-to-n	−0.06	−0.01	−0.11^***^	−0.02	−0.08^**^	−0.02	−0.11^***^	−0.04	0.09^***^	−0.13^***^	−0.03	−0.02
4. Seg. pref. n-to-w	−0.07^**^	0.01	−0.03	−0.09^***^	−0.02	−0.01	0.02	0.07^**^	0.13^***^	−0.02	0.03	−0.02
5. Int. enac. w-to-n	0.15^***^	−0.07^**^	0.12^***^	0.00	0.04	−0.01	0.05	0.06^*^	−0.15^***^	0.30^***^	0.05	0.16^***^
6. Int. enac. n-to-w	0.05	−0.02	0.08^***^	0.03	0.01	0.01	0.03	0.01	−0.16^***^	0.14^***^	0.01	0.14^***^
7. Work role invol.												
8. Nonwork role invol.												
9. Age												
10. Working hours	—											
11. Gender^a^	0.10^*^	—										
12. Education level^b^	0.10^*^	−0.05	—									
13. Partnership^c^	0.08	0.09	−0.04	—								
14. Care for children^c^	−0.02	0.16^**^	0.03	0.25^***^	—							
15. Care for parents^c^	−0.03	0.04	−0.13^**^	0.06	0.07	—						
16. Self-employment^c^	0.12^*^	0.13^**^	0.00	−0.02	−0.04	0.01	—					
17. Supervisory role^c^	0.17^***^	0.16^***^	0.07	0.11^*^	0.11^*^	0.07	0.17^***^	—				
18. Org. tenure	−0.07	0.21^***^	−0.15^**^	0.03	−0.05	0.18^***^	0.11^*^	0.15^**^	—			
19. Availability	0.20^***^	0.00	0.08	0.09	0.11^*^	0.07	0.20^***^	0.20^***^	−0.11^*^	—		
20. Telework	0.02	0.03	0.26^***^	−0.09	−0.04	−0.02	0.29^***^	0.04	0.03	0.08	—	
21. COVID-19 impact	0.02	−0.07	0.15^**^	0.15^**^	0.06	0.11^*^	−0.01	0.03	−0.02	0.19^***^	0.17^***^	—

Cronbach’s alpha (reported on the matrix diagonal in parentheses) for day-level variables are mean internal consistencies averaged over all measurement waves. Correlations below the diagonal are based on responses aggregated to the person level (*N* = 425). Correlations above the diagonal are based on responses aggregated to the day level (*N* = 3,238). Missing cases were excluded pairwise. Seg. pref. = segmentation preference; int. enac. = integration enactment; w-to-n = work-to-nonwork; n-to-w = nonwork-to-work; involv. = involvement; org. tenure = organizational tenure.

a Coding: 0 = woman; 1 = man.

b Treated as a numeric variable from 0 = no graduation to 4 = university degree.

c Coding: 0 = no; 1 = yes.

^*^p < 0.05, ^**^p < 0.01, ^***^p < 0.001.

#### 3.2.1. Confirmatory factor analyses

Before testing the hypotheses, we investigated the underlying factor structure of the study variables using multilevel confirmatory factor analyses. To evaluate the model fit, we used several complementary goodness-of-fit indices and their established cut-off values: Values of at least 0.90 for comparative fit index (CFI) and Tucker–Lewis index (TLI) and below 0.10 for root-mean-square error of approximation (RMSEA) and standardized root-mean-square residual (SRMR) indicate an acceptable fit (Hu and Bentler, 1999; Brown, 2006). We tested an 8-factor multilevel model (Level 1: strain in work life, strain in nonwork life, work-to-nonwork segmentation preference, nonwork-to-work segmentation preference, work-to-nonwork integration enactment, nonwork-to-work integration enactment; Level 2: work role involvement, nonwork role involvement). The resulting model fit was satisfactory, *χ*^2^(139) = 1,090.49, *p* < 0.001, CFI = 0.99, TLI = 0.98, RMSEA = 0.05, SRMR_within_ = 0.02, SRMR_between_ = 0.08, and significantly better compared to the model fit of several other alternative models (see Supplementary Table S2). These findings indicate that the variables measured represented distinct latent constructs and provide evidence of construct validity for the segmentation preference and integration enactment measures at the day level.

#### 3.2.2. Mulitlevel modeling analyses

We pre-registered that we plan to control for sociodemographic variables and the lagged criteria (i.e., segmentation preferences and integration enactments measured on the previous day); however, including the lagged criteria led to the problem that we could include only three quarters of the daily questionnaires (i.e., 2,476 instead of 3,238) in the analyses. We decided to report the results of the analyses without the lagged criteria in the following to consider all data obtained. However, the Supplementary Material provides the results of the analyses with the lagged criteria. In general, the results of the analyses with versus without the lagged criteria were the same. However, there were minor differences in the results, which are reported and discussed in the following.

##### 3.2.2.1. Work-to-nonwork segmentation preference

Table 2 presents the multilevel modeling results for work-to-nonwork segmentation preference. The ICC in the null model indicated that 40.6% of the total variance was attributable to within-person variation. The 95% confidence interval (CI) of the within-person variance component, 95% CI [0.83, 0.93], did not include zero, suggesting a significant amount of intra-individual fluctuation in work-to-nonwork segmentation preference and supporting Hypothesis 1-1a.

**Table 2:** Results of multilevel modeling analyses for work-to-nonwork segmentation preference [Study 1].

Effect	Model				
	Null	Control	Random intercept and fixed slope	Random intercept and random slope	Cross-level interaction
Intercept	5.524^***^ (0.058)	5.813^***^ (0.126)	5.779^***^ (0.120)	5.789^***^ (0.118)	5.790^***^ (0.118)
Age		0.009^*^ (0.005)	0.013^**^ (0.004)	0.011^**^ (0.004)	0.011^**^ (0.004)
Education level^a^		−0.131^*^ (0.060)	−0.116^*^ (0.057)	−0.120^*^ (0.056)	−0.120^*^ (0.056)
Self-employment^b^		−0.587^**^ (0.220)	−0.501^*^ (0.210)	−0.532^*^ (0.206)	−0.531^*^ (0.206)
Availability		−0.137^*^ (0.059)	−0.077 (0.060)	−0.087 (0.059)	−0.087 (0.059)
Work role involvement			−0.028 (0.058)	−0.030 (0.057)	−0.024 (0.057)
Nonwork role involvement			0.326^***^ (0.056)	0.326^***^ (0.056)	0.325^***^ (0.056)
Strain work			0.004^***^ (0.001)	0.004^**^ (0.001)	0.004^**^ (0.001)
Strain nonwork			−0.001 (0.001)	−0.002 (0.001)	−0.002 (0.001)
Strain work × work role involv.					−0.001 (0.001)
Strain work × nonwork role involv.					0.000 (0.002)
Strain nonwork × work role involv.					0.001 (0.001)
Strain nonwork × nonwork role involv.					−0.001 (0.001)
Within-person variance	0.877	0.877	0.873	0.777	0.777
Intercept variance	1.281	1.196	1.067	1.081	1.081
Slope variance | strain work				0.0002	0.0002
Slope variance | strain nonwork				0.0001	0.0001
Intercept-slope correlation | strain work				−0.33	−0.33
Intercept-slope correlation | strain nonwork				0.01	−0.01
ICC	0.594				
Deviance	9,798.7	9,772.3	9,714.8	9,613.2	9,609.8
Marginal *R*^2^	—	0.041	0.105	0.107	0.106
Conditional *R*^2^	0.594	0.594	0.597	0.643	0.641

Level-1 *N* = 3,238 and Level-2 *N* = 425. Unstandardized regression coefficients with standard errors in parentheses are reported. Maximum likelihood estimation of model parameters was used. Involv. = involvement; ICC = intraclass correlation coefficient.

a Treated as a numeric variable from 0 = no graduation to 4 = university degree.

b Coding: 0 = no; 1 = yes.

^*^p < 0.05, ^**^p < 0.01, ^***^p < 0.001.

The random intercept and fixed slope model showed that including work and nonwork role involvement and strain in work and nonwork life significantly improved model fit compared to the control model, *χ*^2^(4) = 57.56, *p* < 0.001. Strain in work life was significantly and positively related to work-to-nonwork segmentation preference, supporting Hypothesis 2-1a. Strain in nonwork life was not significantly related to work-to-nonwork segmentation preference, which did not support Hypothesis 3-1a.

Comparing the models with fixed and random slopes revealed that the model fit improved significantly when the slopes of the Level-1 predictors were allowed to vary randomly across Level-2 units, *χ*^2^(5) = 101.58, *p* < 0.001. The random intercept and slope model showed the same effects as the random intercept and fixed slope model.

Finally, the cross-level interaction model did not support Hypotheses 4-1a and 5-1a, which posited interaction effects. Comparing the models revealed that the fit of the model with interaction terms was not significantly better compared to the model without them, *χ*^2^(4) = 3.41, *p* = 0.491. None of the interaction terms were significant predictors.

Multilevel modeling analysis, including the lagged criterion, yielded the same results regarding the hypotheses (see Supplementary Table S3).

##### 3.2.2.2. Nonwork-to-work segmentation preference

Table 3 presents the multilevel modeling results for nonwork-to-work segmentation preference. The ICC in the null model indicated that 42.1% of the total variance was attributable to within-person variation. The 95% CI of the within-person variance component, 95% CI [1.06, 1.17], did not include zero, suggesting a significant amount of intra-individual fluctuation in nonwork-to-work segmentation preference and supporting Hypothesis 1-1b.

**Table 3:** Results of multilevel modeling analyses for nonwork-to-work segmentation preference [Study 1].

Effect	Model				
	Null	Control	Random intercept and fixed slope	Random intercept and random slope	Cross-level interaction
Intercept	4.473^***^ (0.063)	4.709^***^ (0.117)	4.704^***^ (0.117)	4.693^***^ (0.116)	4.692^***^ (0.116)
Age		0.014^*^ (0.006)	0.014^*^ (0.006)	0.014^*^ (0.006)	0.014^*^ (0.006)
Partnership^a^		−0.327^*^ (0.138)	−0.320^*^ (0.139)	−0.306^*^ (0.137)	−0.306^*^ (0.137)
Organizational tenure		0.009 (0.007)	0.010 (0.007)	0.008 (0.009)	0.008 (0.007)
Work role involvement			0.137^*^ (0.061)	0.131^*^ (0.060)	0.137^*^ (0.061)
Nonwork role involvement			0.001 (0.066)	0.005 (0.065)	−0.001 (0.066)
Strain work			0.001 (0.001)	0.002 (0.001)	0.002 (0.001)
Strain nonwork			0.003^*^ (0.001)	0.004 (0.002)	0.004 (0.002)
Strain work × work role involv.					−0.001 (0.001)
Strain work × nonwork role involv.					−0.002 (0.001)
Strain nonwork × work role involv.					−0.001 (0.002)
Strain nonwork × nonwork role involv.					0.002 (0.002)
Within-person variance	1.111	1.111	1.108	0.934	0.935
Intercept variance	1.527	1.442	1.420	1.443	1.443
Slope variance | strain work				0.0001	0.0001
Slope variance | strain nonwork				0.0006	0.0006
Intercept-slope correlation | strain work				−0.09	−0.09
Intercept-slope correlation | strain nonwork				−0.30	−0.30
ICC	0.579				
Deviance	10,541.1	10,519.4	10,505.7	10,329.9	10,327.3
Marginal *R*^2^	—	0.032	0.042	0.040	0.042
Conditional *R*^2^	0.579	0.579	0.580	0.647	0.647

Level-1 *N* = 3,238 and Level-2 *N* = 425. Unstandardized regression coefficients with standard errors in parentheses are reported. Maximum likelihood estimation of model parameters was used. Involv. = involvement; ICC = intraclass correlation coefficient.

a Coding: 0 = no; 1 = yes.

^*^p < 0.05, ^**^p < 0.01, ^***^p < 0.001.

The random intercept and fixed slope model showed that including work and nonwork role involvement and strain in work and nonwork life significantly improved model fit compared to the control model, *χ*^2^(4) = 13.67, *p* = 0.008. Strain in work life was not significantly related to nonwork-to-work segmentation preference, which did not support Hypothesis 2-1b. Strain in nonwork life was significantly and positively related to nonwork-to-work segmentation preference, supporting Hypothesis 3-1b.

Comparing the models with fixed and random slopes revealed that the model fit improved significantly when the slopes of the Level-1 predictors were allowed to vary randomly across Level-2 units, *χ*^2^(5) = 175.87, *p* < 0.001. The random intercept and slope model showed almost the same effects as the random intercept and fixed slope model. However, the effect of strain in nonwork life did not reach significance in this model (*p* = 0.066).

Finally, the cross-level interaction model did not support Hypotheses 4-1b and 5-1b, which posited interaction effects. Comparing the models revealed that the fit of the model with interaction terms was not significantly better compared to the model without them, *χ*^2^(4) = 2.54, *p* = 0.637. None of the interaction terms were significant predictors.

Multilevel modeling analysis, including the lagged criterion, yielded slightly different results regarding the hypotheses (see Supplementary Table S4). First, the effect of strain in nonwork life did not reach significance in the random intercept and fixed slope model (*p* = 0.188). Second, strain in work life was significantly and positively related to nonwork-to-work segmentation preference in the cross-level interaction model (*p* = 0.040).

##### 3.2.2.3. Work-to-nonwork integration enactment

Table 4 presents the multilevel modeling results for work-to-nonwork integration enactment. The ICC in the null model indicated that 47.6% of the total variance was attributable to within-person variation. The 95% CI of the within-person variance component, 95% CI [1.17, 1.29], did not include zero, suggesting a significant amount of intra-individual fluctuation in work-to-nonwork integration enactment and supporting Hypothesis 1-2a.

**Table 4:** Results of multilevel modeling analyses for work-to-nonwork integration enactment [Study 1].

Effect	Model				
	Null	Control	Random intercept and fixed slope	Random intercept and random slope	Cross-level interaction
Intercept	2.851^***^ (0.060)	2.684^***^ (0.120)	2.716^***^ (0.116)	2.735^***^ (0.115)	2.736^***^ (0.115)
Age		−0.015^**^ (0.005)	−0.020^***^ (0.005)	−0.021^***^ (0.005)	−0.021^***^ (0.005)
Working hours		0.035^**^ (0.013)	0.028^*^ (0.012)	0.026^*^ (0.012)	0.026^*^ (0.012)
Education level^a^		0.091 (0.058)	0.073 (0.055)	0.063 (0.055)	0.063 (0.055)
Organizational tenure		−0.005 (0.006)	−0.002 (0.006)	−0.002 (0.006)	−0.002 (0.006)
Availability		0.365^***^ (0.056)	0.285^***^ (0.058)	0.284^***^ (0.058)	0.283^***^ (0.058)
COVID-19 impact		0.170^**^ (0.053)	0.190^***^ (0.051)	0.186^***^ (0.051)	0.186^***^ (0.051)
Work role involvement			0.102 (0.055)	0.098 (0.055)	0.105 (0.055)
Nonwork role involvement			−0.227^***^ (0.055)	−0.231^***^ (0.055)	−0.227^***^ (0.055)
Strain work			0.013^***^ (0.001)	0.013^***^ (0.002)	0.013^***^ (0.002)
Strain nonwork			0.006^***^ (0.001)	0.006^***^ (0.002)	0.006^***^ (0.002)
Strain work × work role involv.					0.002 (0.002)
Strain work × nonwork role involv.					0.001 (0.002)
Strain nonwork × work role involv.					−0.000 (0.002)
Strain nonwork × nonwork role involv.					0.000 (0.002)
Within-person variance	1.227	1.226	1.159	1.043	1.044
Intercept variance	1.352	1.012	0.929	0.945	0.945
Slope variance | strain work				0.0003	0.0003
Slope variance | strain nonwork				0.0002	0.0002
Intercept-slope correlation | strain work				0.19	0.21
Intercept-slope correlation | strain nonwork				0.02	0.01
ICC	0.524				
Deviance	10,777.5	10,670.5	10,478.5	10,401.5	10,399.2
Marginal *R*^2^	—	0.135	0.194	0.191	0.193
Conditional *R*^2^	0.524	0.526	0.553	0.596	0.597

Level-1 *N* = 3,238 and Level-2 *N* = 425. Unstandardized regression coefficients with standard errors in parentheses are reported. Maximum likelihood estimation of model parameters was used. Involv. = involvement; ICC = intraclass correlation coefficient.

a Treated as a numeric variable from 0 = no graduation to 4 = university degree.

^*^p < 0.05, ^**^p < 0.01, ^***^p < 0.001.

The random intercept and fixed slope model showed that including work and nonwork role involvement and strain in work and nonwork life significantly improved model fit compared to the control model, *χ*^2^(4) = 191.97, *p* < 0.001. Strain in work life was significantly and positively related to work-to-nonwork integration enactment, supporting Hypothesis 2-2a. Strain in nonwork life was significantly and positively related to work-to-nonwork integration enactment, contradicting the negative relationship proposed in Hypothesis 3-2a.

Comparing the models with fixed and random slopes revealed that the model fit improved significantly when the slopes of the Level-1 predictors were allowed to vary randomly across Level-2 units, *χ*^2^(5) = 77.04, *p* < 0.001. The random intercept and slope model showed the same effects as the random intercept and fixed slope model.

Finally, the cross-level interaction model did not support Hypotheses 4-2a and 5-2a, which posited interaction effects. Comparing the models revealed that the fit of the model with interaction terms was not significantly better compared to the model without them, *χ*^2^(4) = 2.23, *p* = 0.693. None of the interaction terms were significant predictors.

Multilevel modeling analysis, including the lagged criterion, yielded the same results regarding the hypotheses (see Supplementary Table S5).

##### 3.2.2.4. Nonwork-to-work integration enactment

Table 5 presents the multilevel modeling results for nonwork-to-work integration enactment. The ICC in the null model indicated that 52.4% of the total variance was attributable to within-person variation. The 95% CI of the within-person variance component, 95% CI [1.25, 1.39], did not include zero, suggesting a significant amount of intra-individual fluctuation in nonwork-to-work integration enactment and supporting Hypothesis 1-2b.

**Table 5:** Results of multilevel modeling analyses for nonwork-to-work integration enactment [Study 1].

Effect	Model				
	Null	Control	Random intercept and fixed slope	Random intercept and random slope	Cross-level interaction
Intercept	3.028^***^ (0.057)	2.953^***^ (0.121)	2.972^***^ (0.121)	2.975^***^ (0.120)	2.975^***^ (0.120)
Age		−0.015^**^ (0.005)	−0.017^***^ (0.005)	−0.017^**^ (0.005)	−0.017^**^ (0.005)
Education level^a^		0.041 (0.058)	0.031 (0.058)	0.030 (0.058)	0.030 (0.058)
Organizational tenure		−0.011 (0.006)	−0.009 (0.006)	−0.010 (0.006)	−0.010 (0.006)
Availability		0.143^*^ (0.056)	0.100 (0.060)	0.094 (0.060)	0.093 (0.060)
COVID-19 impact		0.171^**^ (0.054)	0.182^***^ (0.054)	0.181^***^ (0.053)	0.181^***^ (0.053)
Work role involvement			0.047 (0.058)	0.053 (0.058)	0.051 (0.058)
Nonwork role involvement			−0.119^*^ (0.057)	−0.112^*^ (0.057)	−0.117^*^ (0.057)
Strain work			−0.001 (0.001)	−0.000 (0.001)	−0.000 (0.001)
Strain nonwork			0.022^***^ (0.001)	0.022^***^ (0.002)	0.022^***^ (0.002)
Strain work × work role involv.					0.000 (0.001)
Strain work × nonwork role involv.					0.000 (0.002)
Strain nonwork × work role involv.					0.002 (0.002)
Strain nonwork × nonwork role involv.					0.003 (0.002)
Within-person variance	1.318	1.317	1.207	1.071	1.070
Intercept variance	1.195	1.034	1.024	1.043	1.043
Slope variance | strain work				0.0002	0.0001
Slope variance | strain nonwork				0.0004	0.0004
Intercept-slope correlation | strain work				−0.09	−0.10
Intercept-slope correlation | strain nonwork				−0.10	−0.11
ICC	0.476				
Deviance	10,937.6	10,884.3	10,630.6	10,540.3	10,537.5
Marginal *R*^2^	—	0.066	0.114	0.114	0.115
Conditional *R*^2^	0.476	0.477	0.521	0.576	0.577

Level-1 *N* = 3,238 and Level-2 *N* = 425. Unstandardized regression coefficients with standard errors in parentheses are reported. Maximum likelihood estimation of model parameters was used. Involv. = involvement; ICC = intraclass correlation coefficient.

a Treated as a numeric variable from 0 = no graduation to 4 = university degree.

^*^p < 0.05, ^**^p < 0.01, ^***^p < 0.001.

The random intercept and fixed slope model showed that including work and nonwork role involvement and strain in work and nonwork life significantly improved model fit compared to the control model, *χ*^2^(4) = 253.73, *p* < 0.001. Strain in work life was not significantly related to nonwork-to-work integration enactment, which did not support Hypothesis 2-2b. Strain in nonwork life was significantly and positively related to nonwork-to-work integration enactment, supporting Hypothesis 3-2b.

Comparing the models with fixed and random slopes revealed that the model fit improved significantly when the slopes of the Level-1 predictors were allowed to vary randomly across Level-2 units, *χ*^2^(5) = 90.30, *p* < 0.001. The random intercept and slope model showed the same effects as the random intercept and fixed slope model.

Finally, the cross-level interaction model did not support Hypotheses 4-2b and 5-2b, which posited interaction effects. Comparing the models revealed that the fit of the model with interaction terms was not significantly better compared to the model without them, *χ*^2^(4) = 2.81, *p* = 0.589. None of the interaction terms were significant predictors.

Multilevel modeling analysis, including the lagged criterion, yielded the same results regarding the hypotheses (see Supplementary Table S6).

### 3.3. Conclusion of Study 1

The daily diary study demonstrated that segmentation preferences and integration enactments show significant intra-individual fluctuations. These fluctuations are related to strain in work and nonwork life. Strain in work life is associated with preferring more work-to-nonwork segmentation, preferring more nonwork-to-work segmentation, and enacting more work-to-nonwork integration. In contrast, strain in nonwork life is associated with preferring more nonwork-to-work segmentation, enacting more work-to-nonwork integration, and enacting more nonwork-to-work integration. However, it should be noted that the strain effects on nonwork-to-work segmentation preference were not robust and depended on whether we controlled for the lagged criterion.

The data did not show the assumed interaction effects between role involvement and strain on segmentation preferences and integration enactments.

## 4. Study 2: experimental vignette study

A critical limitation of Study 1 is that we cannot interpret the found strain effects causally. In a recent review of the literature on work–family research, Allen and French (2023) called for using more experimental research designs, which allow for tests of direction and causality. Accordingly, we conducted a second Study (Study 2) using experimental vignette methodology (for best practice recommendations, see Aguinis and Bradley, 2014). Here, we experimentally manipulated strain in work and nonwork life to investigate causal effects on participants’ hypothetical segmentation preferences.

### 4.1. Materials and methods of Study 2

#### 4.1.1. Open science, ethical review, and funding

We did not pre-register the experimental vignette study because the underpinning hypotheses were pre-registered as part of Study 1. Data collection received no funding. Ethics approval was not sought for the present study because we considered it less ethically problematic than the reviewed and approved daily diary study (Study 1), given the hypothetical nature of the situation descriptions, the short duration of the survey, and the low involvement of participants. The participants provided their written informed consent to participate in this study.

#### 4.1.2. Participants and procedure

Participants were recruited through the authors’ professional and social contacts using snowball sampling and through research platforms that support data collection (e.g., SurveyCircle^5^). First, participants were informed about the content and procedure of the study, compensation, data protection, voluntariness, and anonymity; and they gave informed consent. After assessing participants’ *actual* segmentation preferences, we randomly assigned the participants to one of the four vignettes developed for this research. Vignettes represented hypothetical situations describing many versus few stressors that typically cause much versus less strain in work and nonwork life. The vignettes can be found in Supplementary Table S7. We manipulated the degree of strain in work and nonwork life, resulting in a 2 (much versus less strain in work life) × 2 (much versus less strain in nonwork life) research design. We asked participants to read the vignette and rate their *hypothetical* segmentation preferences if they were in the situation described (Finch, 1987). Subsequently, we measured participants’ hypothetical strain in work and nonwork life. Finally, sociodemographic variables were assessed. See Supplementary Table S8 for a data transparency table regarding the variables collected in the experimental vignette study but not used in Study 2. Participants were not offered financial compensation for their participation. However, they received a document with scientifically sound information on successful boundary management tactics.

A total of 283 participants completed the study. To be included in the final sample, participants had to speak German, be between 18 and 67 years old (2 exclusions), be employed or self-employed (38 exclusions), and work at least 20 h per week (50 exclusions). Furthermore, we excluded participants from the final sample if they indicated that they could not imagine being in the vignette situation (9 exclusions). Finally, participants who completed the survey exceptionally fast (i.e., a minimum response time of fewer than 100 s) were excluded (3 exclusions).

The final sample consisted of *N* = 181 participants. Of these, 66.5% were women, and the mean age was 32.01 years (*SD* = 11.46, range: 18–64). About 72.0% indicated living in a partnership, 14.9% cared for at least one child under 18 living in the same household, and 8.0% cared for other people in private life (e.g., elderly parents). More than half of the sample (57.7%) had a university degree, 26.3% had a high school diploma or a university of applied sciences entrance qualification, 12.6% had a secondary school leaving certificate, and 3.4% had a secondary modern school qualification. On average, participants worked 36.97 h per week (*SD* = 9.47, range: 20–60) and had worked for their current employer for 6.05 years (*SD* = 9.27, range: 0–43.5). Some participants indicated being self-employed (11.4%), and 20.6% had a supervisory role. Participants worked in a variety of professional industries. The industries most frequently indicated were services (11.0%) and education (10.4%).

#### 4.1.3. Measures

All items were in German. Scales developed in English were translated and back-translated before (Brislin, 1980).

##### 4.1.3.1. Actual segmentation preference

Actual work-to-nonwork and nonwork-to-work segmentation preferences were measured with Janke et al.’s (2014) Work-to-Nonwork Segmentation Preference Scale and Nonwork-to-Work Segmentation Preference Scale. These scales were translated and adapted from Kreiner (2006) and mirror Michel et al.’s (2022) scales used in Study 1. Both subscales were measured with four items. Example items were “I do not like to think about my work life outside my working hours” (actual work-to-nonwork segmentation preference) and “I do not like to think about my nonwork life while I am at work” (actual nonwork-to-work segmentation preference). Participants rated all items on a Likert scale from 1 (*strongly disagree*) to 7 (*strongly agree*). Cronbach’s alpha was 0.92 for actual work-to-nonwork segmentation preference and 0.83 for actual nonwork-to-work segmentation preference.

##### 4.1.3.2. Hypothetical segmentation preference

Hypothetical work-to-nonwork and nonwork-to-work segmentation preferences were measured by adapting the items used to assess actual segmentation preferences (i.e., adding “In this situation” and phrasing the items in the subjunctive). Both subscales were measured with four items. Example items were “In this situation, I would not like to think about my work life outside my working hours” (hypothetical work-to-nonwork segmentation preference) and “In this situation, I would not like to think about my nonwork life while I am at work” (hypothetical nonwork-to-work segmentation preference). Participants rated all items on a Likert scale from 1 (*strongly disagree*) to 7 (*strongly agree*). Cronbach’s alpha was 0.95 for hypothetical work-to-nonwork segmentation preference and 0.94 for hypothetical nonwork-to-work segmentation preference.

##### 4.1.3.3. Hypothetical strain

We measured hypothetical strain in work and nonwork life by adapting the strain items used in Study 1 (i.e., adding “In this situation” and phrasing the items in the subjunctive). Both subscales were measured with three items. Example items were “In this situation, how much strain would you feel in your work life?” (hypothetical strain in work life) and “In this situation, how much strain would you feel in your nonwork life?” (hypothetical strain in nonwork life). Instead of using visual analog scales as in Study 1, participants rated all items on a Likert scale from 1 (*absolutely not*) to 7 (*absolutely*). Cronbach’s alpha was 0.96 for hypothetical strain in work life and 0.95 for hypothetical strain in nonwork life.

#### 4.1.4. Analytic strategy

All analyses were performed using R (version 4.2.0; R Core Team, 2022). In the first step, we checked the experimental design (i.e., manipulation and randomization) by performing independent samples *t*-tests and two-way analyses of variance (ANOVAs). Next, we tested the hypotheses by analyzing the effects of the two vignette factors (a) strain in work life (much versus less) and (b) strain in nonwork life (much versus less) on hypothetical work-to-nonwork and nonwork-to-work segmentation preferences by performing two two-way ANOVAs.

### 4.2. Results of Study 2

Table 6 reports descriptive statistics, reliabilities, and correlations for all variables.

**Table 6:** Descriptive statistics, reliabilities, and correlations for all variables [Study 2].

Variable	*M*	*SD*	1	2	3	4	5	6	7	8	9
1. IV strain work^a^	—	—	—								
2. IV strain nonwork^a^	—	—	−0.06	—							
3. Hyp. strain work	4.41	1.58	0.71^***^	0.14	(0.96)						
4. Hyp. strain nonwork	4.42	1.62	0.11	0.65^***^	0.39^***^	(0.95)					
5. Actual pref. w-to-n	5.54	1.30	0.06	0.10	0.18^*^	0.13	(0.92)				
6. Actual pref. n-to-w	4.46	1.23	−0.04	0.04	−0.01	0.04	0.47^***^	(0.83)			
7. Hyp. pref. w-to-n	5.28	1.54	0.40^***^	0.05	0.47^***^	0.13	0.41^***^	0.25^***^	(0.95)		
8. Hyp. pref. n-to-w	5.01	1.51	0.24^**^	0.35^***^	0.33^***^	0.47^***^	0.25^***^	0.31^***^	0.24^**^	(0.94)	
9. Age	32.01	11.46	−0.10	0.05	−0.10	−0.02	−0.16^*^	−0.12	−0.13	−0.10	—
10. Working hours	36.97	9.47	−0.08	0.01	−0.08	−0.13	−0.16^*^	−0.11	−0.02	−0.09	0.18^*^
11. Gender^b^	—	—	−0.17^*^	−0.11	−0.27^***^	−0.20^**^	−0.22^**^	−0.15^*^	−0.19^*^	−0.22^**^	0.18^*^
12. Education level^c^	—	—	−0.14	−0.04	−0.06	0.02	−0.03	0.02	−0.09	−0.03	0.04
13. Partnership^d^	—	—	−0.10	0.01	−0.10	−0.04	−0.08	−0.08	−0.04	−0.11	0.19^*^
14. Care for children^d^	—	—	0.02	0.08	0.01	0.05	−0.23^**^	−0.17^*^	−0.04	−0.03	0.26^***^
15. Care for parents^d^	—	—	0.05	0.11	0.07	0.11	0.09	0.05	−0.05	0.05	0.21^**^
16. Employment^d^	—	—	0.10	−0.03	0.00	−0.08	0.09	−0.06	0.03	−0.01	−0.14
17. Self-employment^d^	—	—	−0.08	−0.07	−0.07	−0.01	−0.13	−0.03	−0.05	−0.12	0.24^**^
18. Supervisory role^d^	—	—	−0.20^**^	0.01	−0.17^*^	−0.02	−0.26^***^	−0.09	−0.13	−0.14	0.42^***^
19. Org. tenure	6.05	9.27	−0.07	0.05	−0.03	−0.01	−0.10	−0.09	−0.07	0.00	0.74^***^

Variable	10	11	12	13	14	15	16	17	18
1. IV strain work^a^									
2. IV strain nonwork^a^									
3. Hyp. strain work									
4. Hyp. strain nonwork									
5. Actual pref. w-to-n									
6. Actual pref. n-to-w									
7. Hyp. pref. w-to-n									
8. Hyp. pref. n-to-w									
9. Age									
10. Working hours	—								
11. Gender^b^	0.36^***^	—							
12. Education level^c^	−0.05	0.00	—						
13. Partnership^d^	−0.06	0.09	0.00	—					
14. Care for children^d^	−0.07	−0.02	−0.06	0.15^*^	—				
15. Care for parents^d^	0.01	0.03	−0.06	0.14	0.23^**^	—			
16. Employment^d^	−0.19^*^	−0.16^*^	−0.02	−0.03	−0.05	−0.03	—		
17. Self-employment^d^	0.19^*^	0.24^**^	0.05	−0.02	0.00	−0.04	−0.65^***^	—	
18. Supervisory role^d^	0.22^**^	0.24^**^	−0.06	0.16^*^	0.14	−0.05	−0.20^**^	0.31^***^	—
19. Org. tenure	0.15	0.05	−0.06	0.13	0.17^*^	0.02	−0.10	0.09	0.27^***^

*N* = 181. Cronbach’s alpha is reported on the matrix diagonal in parentheses. Missing cases were excluded pairwise. IV = independent variable; hyp. strain = hypothetical strain; actual pref. = actual segmentation preference; hyp. pref. = hypothetical segmentation preference; w-to-n = work-to-nonwork; n-to-w = nonwork-to-work; org. tenure = organizational tenure.

a Coding: 0 = less strain; 1 = much strain.

b Coding: 0 = woman; 1 = man.

c Treated as a numeric variable from 0 = no graduation to 4 = university degree.

d Coding: 0 = no; 1 = yes.

^*^p < 0.05, ^**^p < 0.01, ^***^p < 0.001.

#### 4.2.1. Checking the experimental design

##### 4.2.1.1. Manipulation checks

We performed two independent samples *t*-tests to analyze (a) the effect of the vignette factor strain in work life (much versus less) on hypothetical strain in work life and (b) the effect of the vignette factor strain in nonwork life (much versus less) on hypothetical strain in nonwork life. We applied Welch’s correction due to a violation of the assumption of homogeneity of variance (i.e., significant Levene’s test combined with unequal sample sizes). First, there was a significant difference in scores of hypothetical strain in work life between participants who imagined being in a situation with much (*M* = 5.41, *SD* = 0.89) versus less (*M* = 3.17, *SD* = 1.35) strain in work life, Welch’s *t*(133.34) = 12.85, *p* < 0.001, Cohen’s *d* = 2.00, 95% CI of Cohen’s *d* [1.64, 2.36]. Second, there was a significant difference in scores of hypothetical strain in nonwork life between participants who imagined being in a situation with much (*M* = 5.27, *SD* = 1.12) versus less (*M* = 3.11, *SD* = 1.38) strain in nonwork life, Welch’s *t*(127.61) = 11.06, *p* < 0.001, Cohen’s *d* = 1.76, 95% CI of Cohen’s *d* [1.41, 2.11]. In short, the manipulation checks supported the proper manipulation of both vignette factors.

##### 4.2.1.2. Randomization checks

We performed two two-way ANOVAs to analyze the effects of the two vignette factors (a) strain in work life (much versus less) and (b) strain in nonwork life (much versus less) on actual work-to-nonwork and nonwork-to-work segmentation preferences. For actual work-to-nonwork segmentation preference, the ANOVA yielded nonsignificant main effects of strain in work life, *F*(1, 177) = 0.63, *p* = 0.427, *η*^2^_p_ < 0.01, and strain in nonwork life, *F*(1, 177) = 1.71, *p* = 0.193, *η*^2^_p_ = 0.01, and a nonsignificant interaction effect, *F*(1, 177) = 0.02, *p* = 0.883, *η*^2^_p_ < 0.01. For actual nonwork-to-work segmentation preference, the ANOVA yielded nonsignificant main effects of strain in work life, *F*(1, 177) = 0.14, *p* = 0.704, *η*^2^_p_ < 0.01, and strain in nonwork life, *F*(1, 177) = 0.32, *p* = 0.575, *η*^2^_p_ < 0.01, and a nonsignificant interaction effect, *F*(1, 177) = 0.13, *p* = 0.718, *η*^2^_p_ < 0.01. In short, the randomization checks supported the successful randomization for the participants’ actual segmentation preferences.

#### 4.2.2. Confirmatory factor analyses

Before testing the hypotheses, we investigated the underlying factor structure of the study variables using confirmatory factor analyses. We tested a 6-factor model (actual work-to-nonwork segmentation preference, actual nonwork-to-work segmentation preference, hypothetical work-to-nonwork segmentation preference, hypothetical nonwork-to-work segmentation preference, hypothetical strain in work life, hypothetical strain in nonwork life). The resulting model fit was satisfactory, *χ*^2^(194) = 300.00, *p* < 0.001, CFI = 0.97, TLI = 0.97, RMSEA = 0.06, SRMR = 0.04, and significantly better compared to the fit of several alternative models (see Supplementary Table S9). These findings indicate that the variables measured represented distinct latent constructs.

#### 4.2.3. Analyses of variance

**Figure 1:**
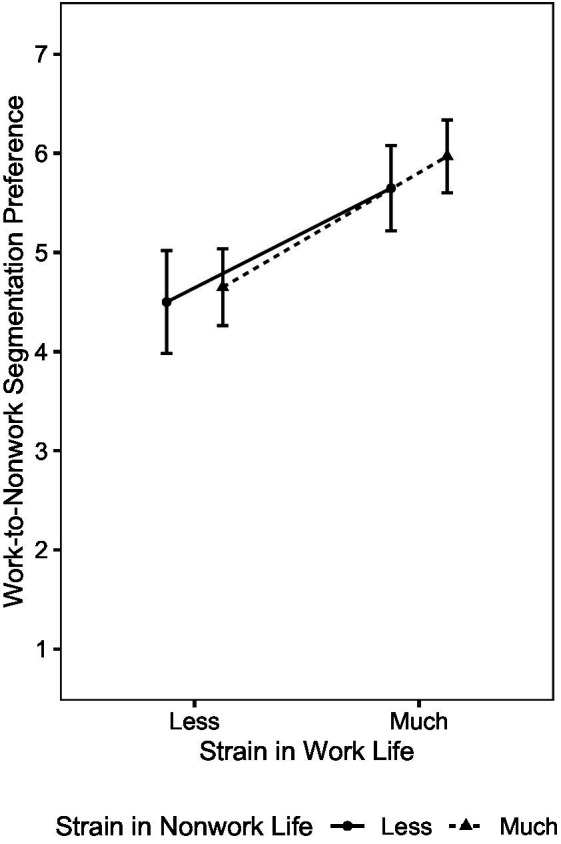
Estimated marginal means with error bars (95% confidence intervals) for work-to-nonwork segmentation preference.

Two two-way ANOVAs were performed to analyze the effects of the two vignette factors (a) strain in work life (much versus less) and (b) strain in nonwork life (much versus less) on hypothetical work-to-nonwork and nonwork-to-work segmentation preferences. For hypothetical work-to-nonwork segmentation preference, the ANOVA yielded a significant main effect of strain in work life, *F*(1, 177) = 32.11, *p* < 0.001, *η*^2^_p_ = 0.15, a nonsignificant main effect of strain in nonwork life, *F*(1, 177) = 1.16, *p* = 0.282, *η*^2^_p_ = 0.01, and a nonsignificant interaction effect, *F*(1, 177) = 0.16, *p* = 0.694, *η*^2^_p_ < 0.01. Figure 1 illustrates the estimated marginal means with error bars. The simple main effect of strain in work life was significant for less strain in nonwork life, mean difference = 1.15, *t*(177) = 3.36, *p* = 0.001, and for much strain in nonwork life, mean difference = 1.32, *t*(177) = 4.89, *p* < 0.001. Participants preferred more work-to-nonwork segmentation in case of much compared to less strain in work life. These results supported Hypothesis 2-1a. The simple main effect of strain in nonwork life was nonsignificant for less strain in work life, mean difference = 0.15, *t*(177) = 0.45, *p* = 0.650, and for much strain in work life, mean difference = 0.32, *t*(177) = 1.12, *p* = 0.265. Participants did not prefer more work-to-nonwork segmentation in case of much compared to less strain in nonwork life. These results did not support Hypothesis 3-1a.

**Figure 2:**
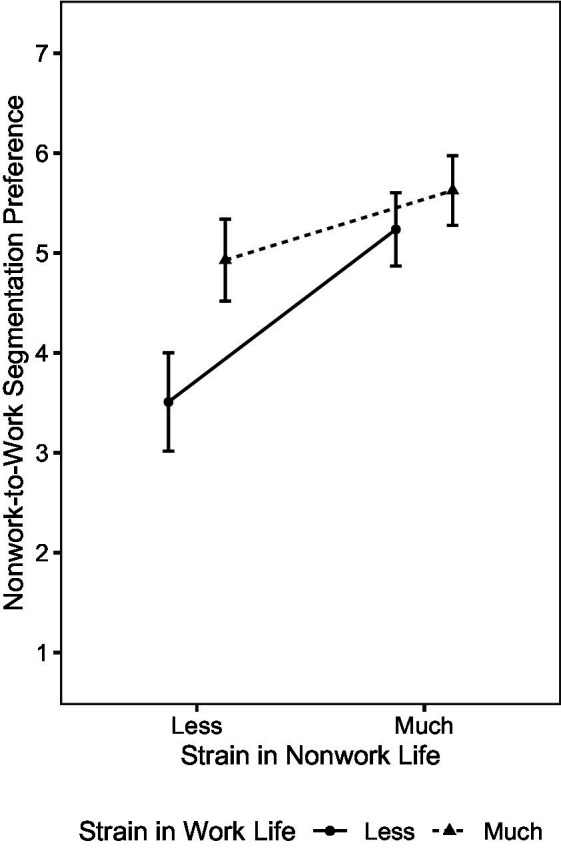
Estimated marginal means with error bars (95% confidence intervals) for nonwork-to-work segmentation preference.

For hypothetical nonwork-to-work segmentation preference, the ANOVA yielded a significant main effect of strain in work life, *F*(1, 177) = 19.13, *p* < 0.001, *η*^2^_p_ = 0.10, a significant main effect of strain in nonwork life, *F*(1, 177) = 34.31, *p* < 0.001, *η*^2^_p_ = 0.16, and a significant interaction effect, *F*(1, 177) = 6.20, *p* = 0.014, *η*^2^_p_ = 0.03. Figure 2 illustrates the estimated marginal means with error bars. The simple main effect of strain in work life was significant for less strain in nonwork life, mean difference = 1.42, *t*(177) = 4.38, *p* < 0.001, but not for much strain in nonwork life, mean difference = 0.39, *t*(177) = 1.52, *p* = 0.131. Participants preferred more nonwork-to-work segmentation in case of much compared to less strain in work life only if there was less strain in nonwork life and not if there was much strain in nonwork life. These results partially supported Hypothesis 2-1b. The simple main effect of strain in nonwork life was significant for less strain in work life, mean difference = 1.73, *t*(177) = 5.55, *p* < 0.001, and for much strain in work life, mean difference = 0.70, *t*(177) = 2.56, *p* = 0.011. Participants preferred more nonwork-to-work segmentation in case of much compared to less strain in nonwork life. These results supported Hypothesis 3-1b.

It should be noted that Levene’s test for homogeneity of variance yielded small *p* values for both analyses (work-to-nonwork segmentation preference: *p* = 0.099; nonwork-to-work segmentation preference: *p* = 0.043). These results, combined with an unbalanced design (i.e., an unequal number of participants across the four vignettes), indicate potential problems due to heteroskedasticity. Therefore, we decided to repeat the analyses with transformed segmentation preference scores. We reflected the scores and applied a square root transformation (see Tabachnick and Fidell, 2014), resolving heteroskedasticity concerns. The results of the ANOVAs with transformed scores replicated those with non-transformed scores (see Supplementary Table S10).

### 4.3. Conclusion of Study 2

The experimental vignette study demonstrated that strain in work and nonwork life causally affect work-to-nonwork and nonwork-to-work segmentation preferences. More specifically, when individuals imagine experiencing much strain in work life, they prefer more work-to-nonwork and nonwork-to-work segmentation than when they imagine experiencing less strain. When individuals imagine experiencing much strain in nonwork life, they prefer more nonwork-to-work segmentation than when they imagine experiencing less strain. These results replicate the findings on the strain effects of Study 1, supplementing them with internally valid data.

## 5. General discussion

In recent years, the concept of boundary management has emerged as an established framework for studying the work–nonwork interface. So far, the focus has been on inter-individual differences in how individuals manage their work–nonwork boundaries and their consequences. In contrast, this paper is one of the first to address intra-individual fluctuations in segmentation preferences and integration enactments and investigate strain levels in work and nonwork life, as well as their interactions with work and nonwork role involvement, as their antecedents. Examining temporal processes in boundary management is critical, given that the work–nonwork interface is dynamic in nature (Grzywacz and Marks, 2000; Ilies et al., 2007; Allen and French, 2023). Furthermore, fluctuations in boundary management have several implications for boundary management theory and its practical application, as outlined below.

We conducted two studies with different methodological approaches. Table 7 gives an overview of the results of the daily diary study (Study 1) and the experimental vignette study (Study 2). The most intriguing finding of Study 1 is that segmentation preferences and integration enactments fluctuate within individuals. Moreover, the results show that experiencing strain in work and nonwork life is related to these daily changes. The strain effects differ for preferences and enactments, such that experiencing more strain is associated with preferring more segmentation but enacting more integration. Study 2 replicated the key results for segmentation preferences and provided evidence for causality. Finally, work and nonwork role involvement were considered person-level moderators of the strain effects. However, the results did not indicate any interaction effect.

**Table 7:** Overview of the results of Study 1 and Study 2.

Effect	Work-to-nonwork segmentation preference			Nonwork-to-work segmentation preference			Work-to-nonwork integration enactment			Nonwork-to-work integration enactment		
	1a	1b	2	1a	1b	2	1a	1b	2	1a	1b	2
Proportion of within-person to total variance	40.6%	38.1%	/	42.1%	38.3%	/	47.6%	46.7%	/	52.4%	49.6%	/
Main effects												
Work strain (1)	+	+	+	o	+^a^	+	+	+	/	o	o	/
Nonwork strain (2)	o	o	o	+^b^	o	+	+^c^	+^c^	/	+	+	/
Work role involvement (3)	o	o	/	+	o	/	o	o	/	o	o	/
Nonwork role involvement (4)	+	+	/	o	o	/	−	−	/	−	o	/
Interaction effects												
(1) × (3)	o	o	/	o	o	/	o	o	/	o	o	/
(1) × (4)	o	o	/	o	o	/	o	o	/	o	o	/
(2) × (3)	o	o	/	o	o	/	o	o	/	o	o	/
(2) × (4)	o	o	/	o	o	/	o	o	/	o	o	/

1a = Study 1 analyses without lagged criterion (reported in the Results section); 1b = Study 1 analyses with lagged criterion (reported in the Supplementary Material); 2 = Study 2 analyses; plus sign (+) = significant and positive effect; minus sign (−) = significant and negative effect; o = nonsignificant effect; slash (/) = not investigated in Study 2.

a The effect was only significant in the cross-level interaction model.

b The effect was only significant in the random intercept and fixed slope model.

c We hypothesized the reversed direction.

### 5.1. Main findings: answering the research questions

#### 5.1.1. Daily fluctuations in preferences and enactments

The first research question addressed daily within-person fluctuations in segmentation preferences and integration enactments. This research question can be answered by stating that segmentation preferences and integration enactments fluctuate daily on an intra-individual level. Consequently, our findings build on Ashforth et al.’s (2000) boundary theory and Clark’s (2000) work/family border theory, which conceptualize work–nonwork boundaries as dynamic and boundary management as a daily experience.

Reviewing the few diary studies published in the boundary management literature (van Steenbergen et al., 2018; Delanoeije et al., 2019; Hecht et al., 2022) revealed that integration enactments show substantial variations within individuals. Our daily diary study results are consistent with these studies, indicating that up to 50% of the total variance in integration enactments results from within-person variations. These findings strengthen Ammons’s (2013) proposition that work–nonwork boundaries progressively change. In contrast, they are inconsistent with the findings reported by Hecht and Allen (2009), who concluded that “work–nonwork boundaries are … relatively stable over time” (p. 853). However, Hecht and Allen (2009) used a longitudinal field study with two measurements separated by 1 year. Thus, the contradictory findings may result from differences in the investigated time horizon.

Moreover, our results provide evidence of significant within-person variations in segmentation preferences. More specifically, around 40% of the total variance in segmentation preferences results from within-person variations. These findings challenge previous notions of segmentation preferences as stable over 1 week (Derks et al., 2016). Consequently, researchers should characterize segmentation preferences as—at least partially—dynamic, given that they can fluctuate in response to changing factors in work and nonwork life. To our knowledge, no other study has addressed such daily fluctuations in segmentation preferences.

Taken together, the findings on intra-individual fluctuations underpin whole trait theory (Fleeson and Jayawickreme, 2015), according to which traits vary as a consequence of variability in situations and experiences. Consequently, it is worth addressing the daily micro-level of boundary management, comprising daily decisions, intentions, and actions regarding managing work–nonwork boundaries (Ashforth et al., 2000; Clark, 2000). As such decisions, intentions, and actions can change in response to internal and external factors, boundary management will likely vary from day to day.

#### 5.1.2. Effects of strain in work and nonwork life

The second research question asked whether strain influences segmentation preferences and integration enactments. It can be answered by stating that strain in work and nonwork life is positively related to segmentation preferences and integration enactments. As shown in Table 7, our results revealed that (a) strain in work life is associated with work-to-nonwork segmentation preference, nonwork-to-work segmentation preference, and work-to-nonwork integration enactment, and (b) strain in nonwork life is associated with nonwork-to-work segmentation preference, work-to-nonwork integration enactment, and nonwork-to-work integration enactment. All these relationships are positive, indicating that individuals prefer more segmentation but enact more integration when they experience high strain levels.

A surprising finding is the positive relationship between strain in nonwork life and work-to-nonwork integration enactment. According to our hypotheses, this relationship should have been negative. We cannot determine the direction of this effect because we did not consider integration enactments in the experimental vignette study. Consequently, the positive relationship could be explained by reversing the hypothesized direction of the effect: Participants could have experienced more strain in nonwork life because they integrated work into nonwork life. Wepfer et al. (2018) outlined similar reasons, suggesting that enacting integration causes strain reactions.

What stands out is that individuals prefer more segmentation when they experience or imagine high strain levels. This finding aligns with results of Kreiner (2006), who showed a significant and positive relationship between segmentation preference and stress. Preferring more segmentation when feeling strained could reflect individuals’ motivation to prevent negative cross-role spillover from the strain-inducing life domain into another (Grzywacz and Marks, 2000; Wepfer et al., 2018). As derived from Hobfoll’s (1989, 2001) conservation of resources theory and ten Brummelhuis and Bakker’s (2012) work–home resources model, they may do so to prevent resource loss associated with strain-induced and negative cross-role spillover. Consequently, preferring more segmentation on days when individuals experience more strain is likely to reflect a functional regulatory reaction (Wepfer et al., 2018).

Contrary to their segmentation preferences, individuals who experience high strain levels tend to integrate work and nonwork life. At first glance, this effect is counter-intuitive because previous research has shown that individuals’ preferences strongly predict corresponding boundary management behavior (Matthews et al., 2010; Methot and LePine, 2016; Palm et al., 2020). Nevertheless, the finding may reflect a critical feature of strain, namely having lower control over one’s enactments than preferences. The job demands–control model (Karasek, 1979) suggests that strain arises from the combined effects of high work demands and low control (Baker, 1985). For example, employees experiencing strain in work life due to a demanding work project (i.e., high work demands) and insufficient time to complete this project (i.e., low control) may feel compelled to take their work home. Consequently, they must integrate work into nonwork life, even though they might prefer segmentation.

#### 5.1.3. The moderating role of work and nonwork role involvement

The third research question asked whether the strain effects on segmentation preferences and integration enactments are the same for all individuals. Our multilevel modeling analyses showed that the strain effects vary across individuals. This finding suggests the need to consider factors that explain slope variances. Hecht et al. (2022) assumed that personal characteristics (e.g., role salience) cause some individuals to experience more or less daily changes in their boundary management than others. Similarly, we decided to consider work and nonwork role involvement as moderators. We elaborated on the theoretical rationales used to justify the strain effects on segmentation preferences and integration enactments (Hobfoll, 1989, 2001; Grzywacz and Marks, 2000; ten Brummelhuis and Bakker, 2012) by taking the strengthening or weakening effect of role involvement into account. As a result, we predicted that individuals more involved in work or nonwork life are less or more likely to adjust their segmentation preferences and integration enactments as a response to experiencing strain compared to individuals less involved.

However, the findings of our daily diary study did not show any evidence of moderation of the strain effects by work and nonwork role involvement. On the one hand, statistical and methodological reasons could explain these null findings. For example, Mathieu et al. (2012) concluded from their large-scale simulation study that “the power to detect cross-level interactions is severely limited in many circumstances” (p. 962) and that “researchers should exercise caution when interpreting statistically nonsignificant cross-level interaction tests” (p. 961). They recommend increasing the lower-level rather than the upper-level sample size with an optimal ratio of 3 to 2 to enhance the power of cross-level interaction tests. In contrast, the Level-2 sample size (i.e., participants) is much larger than the Level-1 sample size (i.e., days) in typical daily diary studies. Moreover, Mathieu et al. (2012) suggest that constraints in the variance of moderators can diminish the magnitude of cross-level interaction effects, which might be the case in our daily diary study.

Besides such statistical and methodological reasons, another explanation for the null findings may be that role involvement varies daily and that this daily or state role involvement affects boundary management. To our knowledge, only one study has examined role involvement as both a trait and a state. In their experience sampling and diary study, Williams and Alliger (1994) distinguished between individuals’ global involvement in work and nonwork roles and daily measures of perceived role involvement. Interestingly, this study also did not find evidence of moderation by the trait measures but revealed direct effects of state role involvement on the work–nonwork interface. Similar to Williams and Alliger’s (1994) reasoning, an alternative explanation for the nonsignificant interaction effects could be that the role involvement measures did not capture the latent characteristics that were expected to moderate the strain effects. Perhaps, it is not a measure of involvement but of identity salience (Capitano and Greenhaus, 2018) or identity centrality (Kossek and Lautsch, 2012) that moderates the strain effects. Similarly, Clark (2000) argues that role involvement is distinct from role identification, which may exhibit a more substantial moderation effect. Altogether, we need a deeper understanding of the underlying constructs and their variations over time to address the research question about the moderation of the strain effects.

### 5.2. Theoretical implications and future research directions

The findings described in this paper have several implications for boundary management theory and research. In general, the two studies enrich our understanding of temporal dynamics in boundary management and their driving factors.

Much of the existing literature has treated boundary management preferences as dispositional traits, leading to the emergence of the trait-like terms *integrator* versus *segmentor* for describing individuals (Nippert-Eng, 1996; Ashforth et al., 2000; Rothbard and Ollier-Malaterre, 2016). Given the considerable amount of within-person variation in segmentation preferences and integration enactments, theories on boundary management should consider that the way people (prefer to) manage work and nonwork life differs between and within individuals. Consequently, the boundary management field may profit from considering a multilevel approach of boundary management with at least two levels: a between-person level, addressing inter-individual differences, and a within-person level, reflecting intra-individual changes. More specifically, we propose that individuals have different baseline levels of segmentation preferences and integration enactments (i.e., *trait component*), representing a personal equilibrium (Smith et al., 2022). However, individuals’ daily segmentation preferences and integration enactments vary around those baseline levels (i.e., *state component*), reflecting fluctuations around the personal equilibrium (Smith et al., 2022).

This multilevel approach mirrors the dynamic framework of boundary permeability recently introduced by Hecht et al. (2022). It surpasses the validity of solely static and dynamic models (Smith et al., 2022) and accommodates Bakker’s (2015) request to adopt a multilevel perspective when investigating constructs in work and organizational psychology. Furthermore, it has the potential to provide novel insights and open several new research questions. For example, when applying the two-level model to the current concept of segmentation preferences, future research might differentiate general or baseline segmentation *preferences* as more stable traits from the segmentation *motivations* or *intentions*, reflecting more volatile states. Besides, scholars could introduce a third level by examining, for example, segmentation *norms* at the group, family, or team level. Altogether, our results provide a significant first step towards a more nuanced understanding of boundary management on different levels.

Furthermore, changes in segmentation preferences and integration enactments suggest that the congruence between these variables could also fluctuate. The current boundary management literature agrees that the experience of fit plays a critical role in determining several outcomes (Chen et al., 2009; Bogaerts et al., 2018). Given that the fit concept has become an influential framework (Ammons, 2013; Bogaerts et al., 2018; Capitano and Greenhaus, 2018; Michel et al., 2022; Mueller and Kempen, 2022), research on daily fluctuations in boundary management fit could reveal findings with high relevance for theory and practice. For example, intra-individual fluctuations in fit (i.e., state component of fit) might affect other outcomes than inter-individual differences in baseline fit levels (i.e., trait component of fit).

Another implication is that the boundary management literature should address not only the consequences but also the antecedents of boundary management. Examples are Capitano et al. (2017) and Palm et al. (2020), who examined antecedents of segmentation preferences (e.g., role salience) and integration enactments (e.g., norms), respectively. Whereas these studies addressed antecedents at the between-person level, the present study is the first that explicitly addresses antecedents at the within-person level. Consequently, we respond to van Steenbergen et al.’s (2018) call to identify factors that trigger individuals to segment or integrate today more or less than tomorrow. Beyond strain-related antecedents, other factors could also affect individuals’ boundary management. For example, positive and negative affective states in work and nonwork life might influence segmentation preferences (Mueller et al., 2022a). Altogether, future research could address other personal and situational antecedents of intra-individual fluctuations in boundary management constructs (Smith et al., 2022).

Next, the association between strain in work life and work-to-nonwork segmentation preference might explain why most studies found a strong segmentation preference in the population of professionals (Allen et al., 2021; Michel et al., 2022). Assuming that many individuals experience high levels of work strain in the modern world of work (Reif et al., 2021), they should prefer to segment nonwork life from work intrusions. It could be interesting to investigate segmentation preferences in individuals with relatively low strain levels or to control for strain when investigating segmentation preferences.

Furthermore, we support Hecht et al.’s (2022) call that research should address cross-level moderators of daily relationships. Although the present study did not find moderation by work or nonwork role involvement, future studies could, for example, examine personal resources such as resilience or coping skills as moderators. Beyond the person level, characteristics of the environment could also explain the differential effects. For example, external resources such as employees’ job autonomy or control over their work–nonwork interface could be situational moderators. Here, the work–home resources model by ten Brummelhuis and Bakker (2012) may provide starting points for interesting research questions.

Finally, the present study shows that the strain effects differ between segmentation preferences and integration enactments. These differences are worth highlighting, as they suggest two crucial points. First, experiencing negative states in a life domain appears to be accompanied by the intention to protect other life domains from possible negative cross-role spillover. Second, individuals cannot transfer their intentions (e.g., segmentation preferences) into behaviors (e.g., segmentation enactments). Ajzen’s (1991) theory of planned behavior addresses such intention–behavior gaps and may provide interesting starting points for future research.

### 5.3. Practical implications

The findings of our studies have several practical implications for organizations and individuals. First, this work demonstrates that segmentation preferences fluctuate from day to day. For this reason, organizations should provide boundary management supplies that account for not only inter-individual differences (Bogaerts et al., 2018; Piszczek and Berg, 2020) but also intra-individual fluctuations in segmentation preferences. For example, organizations can help employees craft their work in ways that meet their preferences by providing daily flexibility and choice. As a result, the daily fit between preferences and supplies should increase, producing a variety of positive consequences (Chen et al., 2009; Bogaerts et al., 2018; Mueller and Kempen, 2022).

Another promising application might build on the finding that strain affects segmentation preferences and integration enactments differentially. Organizations should be aware of their potential influence on their employees’ boundary management via decreasing or increasing strain in work life. For example, the higher the strain level in work life, the more employees prefer segmentation but enact integration. This divergence in preferences and enactments should result in a perceived misfit between desired and enacted boundaries (Ammons, 2013; Capitano and Greenhaus, 2018), which is associated with adverse effects (Michel et al., 2022).

Finally, our findings can help design training and coaching programs at the individual level. For instance, coaches could support individuals in reflecting on their segmentation preferences and integration enactments and monitoring daily changes in these variables. So, they could learn how to use boundary management adaptively to achieve positive and prevent negative work–nonwork spillover (Rexroth et al., 2016; Althammer et al., 2021). In resilience workshops, employees may acquire strategies and resources to better cope with strain in work and nonwork life and maintain a high fit between their preferences and behaviors despite the strain.

### 5.4. Limitations

Despite some notable strengths of the current paper, it has some limitations, which should be considered when interpreting the results. First, we measured all constructs using self-reports of the same person. Consequently, common method bias may have affected the observed relationships (Podsakoff et al., 2003). However, all constructs represented internal variables, which can be best measured by asking people directly. Furthermore, we removed inter-individual differences in the daily diary study by person-mean centering the predictors and focusing on intra-individual differences (Sonnentag et al., 2008).

A related limitation of our daily diary study is that it did not separate the measurement of the predictor and outcome variables. This methodological decision has led to difficulties in interpreting the direction of the found relationships, and the possibility of reversed causality must be considered. To accommodate this limitation, we conducted another study using an experimental research design. We decided to address segmentation preferences rather than integration enactments in our experimental vignette study because participants might be better able to report their preferences than their behaviors in hypothetical vignette situations. Therefore, future research should address the question of causality for the strain effects on integration enactments. Furthermore, diary studies with more than one measurement wave per day (e.g., assessing strain in work life at the end of the workday and work-to-nonwork segmentation preference before going to bed) could yield new insights that improve the accuracy and validity of the present research.

Finally, the present paper focused on short-term (i.e., daily) variations in segmentation preferences and integration enactments as a function of daily strain levels. However, these state components in preferences and enactments should coexist with trait components, representing baseline levels over the longer run (Hecht et al., 2022; Smith et al., 2022). Longitudinal studies over several months or years are needed to capture long-term changes in these trait components, which may occur in response to major life events (e.g., the birth of a child; Allen et al., 2019; Smith et al., 2022). Thus, researchers should address state and trait components by combining daily diary and longitudinal studies.

## 6. Conclusion

We aimed to deepen our understanding of temporal dynamics in boundary management and their antecedents. Our research demonstrates that segmentation preferences and integration enactments differ not only on the inter-individual level but also on the intra-individual level, questioning assumptions in the literature that, primarily, segmentation preferences are highly stable. Moreover, we showed that strain in work and nonwork life are antecedents of these daily fluctuations. More specifically, strain increases segmentation preferences and integration enactments. These results may stimulate future research by representing a starting point for establishing a multilevel model of boundary management and its antecedents.

## Data availability statement

The datasets presented in this study can be found in online repositories. The names of the repository/repositories and accession number(s) can be found at: daily diary study (Study 1): PsychArchives (https://doi.org/10.23668/psycharchives.8139); experimental vignette study (Study 2): PsychArchives (https://doi.org/10.23668/psycharchives.12372).

## Ethics statement

The daily diary study (Study 1) involving human participants was reviewed and approved by the Ethics Committee of the University of Osnabrück. The patients/participants provided their written informed consent to participate in this study. The experimental vignette study (Study 2) involving human participants was not reviewed by a formal ethics committee because the authors considered it less ethically problematic than the reviewed and approved daily diary study (Study 1), given the hypothetical nature of the situation descriptions, the short duration of the survey, and the low involvement of participants. The patients/participants provided their written informed consent to participate in this study.

## Author contributions

NM developed the research idea and conceptual framework, pre-registered the daily diary study (Study 1), contributed to the conception and design of the studies, performed the statistical analysis, and wrote the first draft of the manuscript. SL helped to pre-register the daily diary study (Study 1) and collected the data for the daily diary study (Study 1). EV collected the data for the experimental vignette study (Study 2). RK supervised all steps of the research process, provided critical revisions, and edited the manuscript. All authors contributed to the article and approved the submitted version.

## Footnotes

^1^We chose a consistent nomenclature for organizing the hypotheses and analyses. Specifically, hypotheses with the suffixes -1 and -2 refer to segmentation preferences and integration enactments, respectively. The letters (a) and (b) refer to the directions work-to-nonwork and nonwork-to-work, respectively.

^2^The pre-registration of Study 1 also reports the main effects of work and nonwork role involvement on segmentation preferences and integration enactments. However, the present article does not address these main effects for the sake of brevity and conciseness. First, these effects are already known in the boundary management literature. Second, these effects should occur at the between-person level, but the article focuses on the within-person level.

^3^https://prereg-psych.org/index.php/rrp/calls

^4^https://www.respondi.com/EN/academics

^5^https://www.surveycircle.com

## References

[ref1] Aguinis H. Bradley K. J. (2014). Best practice recommendations for designing and implementing experimental vignette methodology studies. Organ. Res. Methods 17, 351–371. doi: 10.1177/1094428114547952

[ref2] Aguinis H. Gottfredson R. K. Culpepper S. A. (2013). Best-practice recommendations for estimating cross-level interaction effects using multilevel modeling. J. Manag. 39, 1490–1528. doi: 10.1177/0149206313478188

[ref1004] Ajzen I. (1991). The theory of planned behavior. Organ. Behav. Hum. Decis. Process. 50, 179–211. doi: 10.1016/0749-5978(91)90020-T

[ref3] Allen T. D. Cho E. Meier L. L. (2014). Work-family boundary dynamics. Annu. Rev. Organ. Psychol. Organ. Behav. 1, 99–121. doi: 10.1146/annurev-orgpsych-031413-091330

[ref4] Allen T. D. French K. A. (2023). Work-family research: a review and next steps. Pers. Psychol. doi: 10.1111/peps.12573. [E-pub ahead of print].

[ref5] Allen T. D. French K. A. Braun M. T. Fletcher K. (2019). The passage of time in work-family research: toward a more dynamic perspective. J. Vocat. Behav. 110, 245–257. doi: 10.1016/j.jvb.2018.11.013

[ref6] Allen T. D. Merlo K. Lawrence R. C. Slutsky J. Gray C. E. (2021). Boundary management and work-nonwork balance while working from home. Appl. Psychol. 70, 60–84. doi: 10.1111/apps.12300

[ref7] Althammer S. E. Reis D. van der Beek S. Beck L. Michel A. (2021). A mindfulness intervention promoting work–life balance: how segmentation preference affects changes in detachment, well-being, and work–life balance. J. Occup. Organ. Psychol. 94, 282–308. doi: 10.1111/joop.12346

[ref8] Ammons S. K. (2013). Work-family boundary strategies: stability and alignment between preferred and enacted boundaries. J. Vocat. Behav. 82, 49–58. doi: 10.1016/j.jvb.2012.11.002, PMID: 25620801 PMC4303250

[ref9] Ashforth B. E. Kreiner G. E. Fugate M. (2000). All in a day’s work: boundaries and micro role transitions. AMR 25, 472–491. doi: 10.5465/amr.2000.3363315

[ref10] Baker D. B. (1985). The study of stress at work. Annu. Rev. Public Health 6, 367–381. doi: 10.1146/annurev.pu.06.050185.002055 3994817

[ref11] Bakker A. B. (2015). Towards a multilevel approach of employee well-being. Eur. J. Work. Organ. 24, 839–843. doi: 10.1080/1359432X.2015.1071423

[ref12] Bakker A. B. Du D. Derks D. (2019). Major life events in family life, work engagement, and performance: a test of the work-home resources model. Int. J. Stress. Manag. 26, 238–249. doi: 10.1037/str0000108

[ref13] Bates D. Mächler M. Bolker B. Walker S. (2015). Fitting linear mixed-effects models using lme4. J. Stat. Softw. 67, 1–48. doi: 10.18637/jss.v067.i01

[ref14] Bogaerts Y. De Cooman R. De Gieter S. (2018). Getting the work-nonwork interface you are looking for: the relevance of work-nonwork boundary management fit. Front. Psychol. 9:1158. doi: 10.3389/fpsyg.2018.01158, PMID: 30065680 PMC6057117

[ref15] Boswell W. R. Olson-Buchanan J. B. (2007). The use of communication technologies after hours: the role of work attitudes and work-life conflict. J. Manag. 33, 592–610. doi: 10.1177/0149206307302552

[ref16] Brantley P. J. Cocke T. B. Jones G. N. Goreczny A. J. (1988). The daily stress inventory: validity and effect of repeated administration. J. Psychopathol. Behav. Assess. 10, 75–81. doi: 10.1007/BF00962987

[ref17] Brislin R. W. (1980). “Translation and content analysis of oral and written material” in Handbook of cross-cultural psychology. eds. Triandis H. C. Berry J. W. (Boston, MA: Allyn & Bacon), 389–444.

[ref18] Brown T. A. (2006). Confirmatory factor analysis for applied research. New York: Guilford Press.

[ref19] Bulger C. A. Matthews R. A. Hoffman M. E. (2007). Work and personal life boundary management: boundary strength, work/personal life balance, and the segmentation-integration continuum. J. Occup. Health Psychol. 12, 365–375. doi: 10.1037/1076-8998.12.4.365, PMID: 17953495

[ref20] Buss A. R. (1977). The trait-situation controversy and the concept of interaction. Personal. Soc. Psychol. Bull. 3, 196–201. doi: 10.1177/014616727700300207

[ref21] Capitano J. DiRenzo M. S. Aten K. J. Greenhaus J. H. (2017). Role identity salience and boundary permeability preferences: an examination of enactment and protection effects. J. Vocat. Behav. 102, 99–111. doi: 10.1016/j.jvb.2017.07.001

[ref22] Capitano J. Greenhaus J. H. (2018). When work enters the home: antecedents of role boundary permeability behavior. J. Vocat. Behav. 109, 87–100. doi: 10.1016/j.jvb.2018.10.002

[ref23] Carlson D. S. Kacmar K. M. Zivnuska S. Ferguson M. (2015). Do the benefits of family-to-work transitions come at too great a cost? J. Occup. Health Psychol. 20, 161–171. doi: 10.1037/a0038279, PMID: 25365628

[ref24] Chen Z. Powell G. N. Greenhaus J. H. (2009). Work-to-family conflict, positive spillover, and boundary management: a person-environment fit approach. J. Vocat. Behav. 74, 82–93. doi: 10.1016/j.jvb.2008.10.009

[ref25] Cho S. Kim S. Chin S. W. Ahmad U. (2020). Daily effects of continuous ICT demands on work–family conflict: negative spillover and role conflict. Stress. Health 36, 533–545. doi: 10.1002/smi.2955, PMID: 32374072

[ref26] Clark S. C. (2000). Work/family border theory: a new theory of work/family balance. Hum. Relat. 53, 747–770. doi: 10.1177/0018726700536001

[ref27] De Gieter S. Hofmans J. Bakker A. B. (2018). Need satisfaction at work, job strain, and performance: a diary study. J. Occup. Health Psychol. 23, 361–372. doi: 10.1037/ocp0000098, PMID: 28836801

[ref28] Delanoeije J. Verbruggen M. Germeys L. (2019). Boundary role transitions: a day-to-day approach to explain the effects of home-based telework on work-to-home conflict and home-to-work conflict. Hum. Relat. 72, 1843–1868. doi: 10.1177/0018726718823071

[ref29] Derks D. Bakker A. B. Peters P. van Wingerden P. (2016). Work-related smartphone use, work–family conflict and family role performance: the role of segmentation preference. Hum. Relat. 69, 1045–1068. doi: 10.1177/0018726715601890

[ref30] Desrochers S. Hilton J. M. Larwood L. (2005). Preliminary validation of the work-family integration-blurring scale. J. Fam. Issues 26, 442–466. doi: 10.1177/0192513X04272438

[ref31] Eby L. T. Casper W. J. Lockwood A. Bordeaux C. Brinley A. (2005). Work and family research in IO/OB: content analysis and review of the literature (1980–2002). J. Vocat. Behav. 66, 124–197. doi: 10.1016/j.jvb.2003.11.003

[ref32] Edwards J. R. Rothbard N. P. (2000). Mechanisms linking work and family: clarifying the relationship between work and family constructs. AMR 25, 178–199. doi: 10.2307/259269

[ref33] Field J. C. Chan X. W. (2018). Contemporary knowledge workers and the boundaryless work–life interface: implications for the human resource management of the knowledge workforce. Front. Psychol. 9:2414. doi: 10.3389/fpsyg.2018.02414, PMID: 30555399 PMC6283975

[ref1003] Finch J. (1987). The vignette technique in survey research. Sociology 21, 105–114. doi: 10.1177/0038038587021001008

[ref34] Fleeson W. Jayawickreme E. (2015). Whole trait theory. J. Res. Pers. 56, 82–92. doi: 10.1016/j.jrp.2014.10.009, PMID: 26097268 PMC4472377

[ref35] Foucreault A. Ollier-Malaterre A. Ménard J. (2018). Organizational culture and work–life integration: a barrier to employees’ respite? Int. J. Hum. Resour. Manag. 29, 2378–2398. doi: 10.1080/09585192.2016.1262890

[ref36] Frone M. R. Rice R. W. (1987). Work-family conflict: the effect of job and family involvement. J. Organ. Behav. 8, 45–53. doi: 10.1002/job.4030080106

[ref37] Glavin P. Schieman S. (2012). Work–family role blurring and work–family conflict: the moderating influence of job resources and job demands. Work. Occup. 39, 71–98. doi: 10.1177/0730888411406295

[ref38] Greenhaus J. H. Beutell N. J. (1985). Sources of conflict between work and family roles. AMR 10, 76–88. doi: 10.2307/258214

[ref39] Greenhaus J. H. Powell G. N. (2006). When work and family are allies: a theory of work-family enrichment. AMR 31, 72–92. doi: 10.2307/20159186

[ref40] Griffin M. A. Clarke S. (2011). “Stress and well-being at work” in APA handbook of industrial and organizational psychology, Vol 3: maintaining, expanding, and contracting the organization. ed. Zedeck S. (Washington, DC: American Psychological Association), 359–397. doi: 10.1037/12171-010

[ref41] Grzywacz J. G. Marks N. F. (2000). Reconceptualizing the work–family interface: an ecological perspective on the correlates of positive and negative spillover between work and family. J. Occup. Health Psychol. 5, 111–126. doi: 10.1037/1076-8998.5.1.111, PMID: 10658890

[ref42] Hecht T. D. Allen N. J. (2009). A longitudinal examination of the work–nonwork boundary strength construct. J. Organ. Behav. 30, 839–862. doi: 10.1002/job.579

[ref43] Hecht T. D. Cluley H. Lefter A. M. Ngamwattana O. A. (2022). A dynamic framework of boundary permeability: daily events and within-individual fluctuations in daily work and nonwork boundary permeation. Eur. J. Work. Organ. 32, 23–46. doi: 10.1080/1359432X.2022.2081075

[ref44] Hirschi A. von Allmen N. Burmeister A. Zacher H. (2022). Action regulation at the work–family interface: Nomological network and work–family consequences. J. Bus. Psychol. 37, 369–387. doi: 10.1007/s10869-021-09751-6

[ref45] Hobfoll S. E. (1989). Conservation of resources: a new attempt at conceptualizing stress. Am. Psychol. 44, 513–524. doi: 10.1037/0003-066X.44.3.513, PMID: 2648906

[ref46] Hobfoll S. E. (2001). The influence of culture, community, and the nested-self in the stress process: advancing conservation of resources theory. Appl. Psychol. 50, 337–421. doi: 10.1111/1464-0597.00062

[ref47] Horvath M. Gueulette J. S. Gray K. A. (2021). Employee reactions to interruptions from family during work. Occup. Health Sci. 5, 141–162. doi: 10.1007/s41542-021-00081-w, PMID: 33816779 PMC7997796

[ref48] Houdmont J. Jachens L. Randall R. Hopson S. Nuttall S. Pamia S. (2019). What does a single-item measure of job stressfulness assess? Int. J. Environ. Health Res. 16:1480. doi: 10.3390/ijerph16091480, PMID: 31027356 PMC6539290

[ref49] Hu L. Bentler P. M. (1999). Cutoff criteria for fit indexes in covariance structure analysis: conventional criteria versus new alternatives. Struct. Equ. Modeling 6, 1–55. doi: 10.1080/10705519909540118

[ref50] Huang J. L. Curran P. G. Keeney J. Poposki E. M. DeShon R. P. (2012). Detecting and deterring insufficient effort responding to surveys. J. Bus. Psychol. 27, 99–114. doi: 10.1007/s10869-011-9231-8

[ref51] Ilies R. Schwind K. M. Wagner D. T. Johnson M. D. DeRue D. S. Ilgen D. R. (2007). When can employees have a family life? The effects of daily workload and affect on work-family conflict and social behaviors at home. J. Appl. Psychol. 92, 1368–1379. doi: 10.1037/0021-9010.92.5.1368, PMID: 17845091

[ref52] Janke I. Stamov-Roßnagel C. Scheibe S. (2014). Verschwimmen die Grenzen? Auswirkungen von Vertrauensarbeitszeit auf die Schnittstelle von Arbeit und Privatleben [Blurring boundaries? The impact of trust-based working time on the work / non-work interface]. Z. Arb. Wiss. 68, 97–104. doi: 10.1007/BF03374430

[ref53] Kanner A. D. Coyne J. C. Schaefer C. Lazarus R. S. (1981). Comparison of two modes of stress measurement: daily hassles and uplifts versus major life events. J. Behav. Med. 4, 1–39. doi: 10.1007/BF00844845, PMID: 7288876

[ref54] Kanungo R. N. (1982). Measurement of job and work involvement. J. Appl. Psychol. 67, 341–349. doi: 10.1037/0021-9010.67.3.341

[ref55] Karasek R. A. (1979). Job demands, job decision latitude, and mental strain: implications for job redesign. Adm. Sci. Q. 24, 285–308. doi: 10.2307/2392498

[ref56] Katz D. Kahn R. L. (1966). The social psychology of organizations. New York: Wiley.

[ref57] Kempen R. Roewekaemper J. Hattrup K. Mueller K. (2019). Daily affective events and mood as antecedents of life domain conflict and enrichment: a weekly diary study. Int. J. Stress. Manag. 26, 107–119. doi: 10.1037/str0000104

[ref58] Kniffin K. M. Narayanan J. Anseel F. Antonakis J. Ashford S. P. Bakker A. B.. (2021). COVID-19 and the workplace: implications, issues, and insights for future research and action. Am. Psychol. 76, 63–77. doi: 10.1037/amp0000716, PMID: 32772537

[ref59] Kossek E. E. Lautsch B. A. (2012). Work–family boundary management styles in organizations: a cross-level model. Organ. Psychol. Rev. 2, 152–171. doi: 10.1177/2041386611436264

[ref60] Kossek E. E. Ruderman M. N. Braddy P. W. Hannum K. M. (2012). Work–nonwork boundary management profiles: a person-centered approach. J. Vocat. Behav. 81, 112–128. doi: 10.1016/j.jvb.2012.04.003

[ref61] Kreiner G. E. (2006). Consequences of work-home segmentation or integration: a person-environment fit perspective. J. Organ. Behav. 27, 485–507. doi: 10.1002/job.386

[ref62] Kreiner G. E. Hollensbe E. C. Sheep M. L. (2009). Balancing borders and bridges: negotiating the work-home interface via boundary work tactics. AMJ 52, 704–730. doi: 10.5465/AMJ.2009.43669916

[ref63] Lewin K. (1935). A dynamic theory of personality. New York: McGraw-Hill.

[ref64] Littman A. J. White E. Satia J. A. Bowen D. J. Kristal A. R. (2006). Reliability and validity of 2 single-item measures of psychosocial stress. Epidemiology 17, 398–403. doi: 10.1097/01.ede.0000219721.89552.51, PMID: 16641618

[ref65] Luhmann M. Hofmann W. Eid M. Lucas R. E. (2012). Subjective well-being and adaptation to life events: a meta-analysis. J. Pers. Soc. Psychol. 102, 592–615. doi: 10.1037/a0025948, PMID: 22059843 PMC3289759

[ref66] Mathieu J. E. Aguinis H. Culpepper S. A. Chen G. (2012). Understanding and estimating the power to detect cross-level interaction effects in multilevel modeling. J. Appl. Psychol. 97, 951–966. doi: 10.1037/a0028380, PMID: 22582726

[ref67] Matthews R. A. Barnes-Farrell J. L. Bulger C. A. (2010). Advancing measurement of work and family domain boundary characteristics. J. Vocat. Behav. 77, 447–460. doi: 10.1016/j.jvb.2010.05.008

[ref68] McNall L. A. Scott L. D. Nicklin J. M. (2015). Do positive affectivity and boundary preferences matter for work–family enrichment? A study of human service workers. J. Occup. Health Psychol. 20, 93–104. doi: 10.1037/a0038165, PMID: 25347683

[ref69] Methot J. R. LePine J. A. (2016). Too close for comfort? Investigating the nature and functioning of work and non-work role segmentation preferences. J. Bus. Psychol. 31, 103–123. doi: 10.1007/s10869-015-9402-0

[ref70] Michel J. S. Rotch M. A. O’Neill S. K. (2022). The effects of work and nonwork boundary fit on role satisfaction and subjective well-being. Stress. Health 38, 163–170. doi: 10.1002/smi.3070, PMID: 34021679

[ref71] Motowidlo S. J. Packard J. S. Manning M. R. (1986). Occupational stress: its causes and consequences for job performance. J. Appl. Psychol. 71, 618–629. doi: 10.1037/0021-9010.71.4.618 3804934

[ref72] Mueller N. Kempen R. (2022). The influence of boundary management preference on work–nonwork policy effectiveness: is “turning off” the solution? Eur. J. Work. Organ. doi: 10.1080/1359432X.2022.2161371. [E-pub ahead of print].

[ref73] Mueller N. Loeffelsend S. Kempen R. (2022a). Effects of daily affective experiences on boundary management preferences–a daily diary study. PsychArchives. doi: 10.23668/PSYCHARCHIVES.5398

[ref1002] Mueller N. Loeffelsend S. Kempen R. (2022b). Effects of daily strain on boundary management preference and enactment – a daily diary study. PsychArchives. doi: 10.23668/PSYCHARCHIVES.5393 PMC10628038 37941752

[ref74] Nakagawa S. Johnson P. C. D. Schielzeth H. (2017). The coefficient of determination *R*^2^ and intra-class correlation coefficient from generalized linear mixed-effects models revisited and expanded. J. R. Soc. Interface 14:20170213. doi: 10.1098/rsif.2017.0213, PMID: 28904005 PMC5636267

[ref75] Nezlek J. B. (2012). “Multilevel modeling for psychologists” in APA handbook of research methods in psychology, Vol 3: Data analysis and research publication. eds. Cooper H. Camic P. M. Long D. L. Panter A. T. Rindskopf D. Sher K. J. (Washington, D.C.: American Psychological Association), 219–241.

[ref76] Nezlek J. B. (2020). Diary studies in social and personality psychology: an introduction with some recommendations and suggestions. Soc. Psychol. Bull. 15:e2679. doi: 10.32872/spb.2679

[ref77] Nippert-Eng C. (1996). Calendars and keys: the classification of “home” and “work”. Sociol. Forum 11, 563–582. doi: 10.1007/BF02408393

[ref78] Ohly S. Sonnentag S. Niessen C. Zapf D. (2010). Diary studies in organizational research: an introduction and some practical recommendations. J. Pers. Psychol. 9, 79–93. doi: 10.1027/1866-5888/a000009

[ref79] Ollier-Malaterre A. Jacobs J. A. Rothbard N. P. (2019). Technology, work, and family: digital cultural capital and boundary management. Annu. Rev. Sociol. 45, 425–447. doi: 10.1146/annurev-soc-073018-022433

[ref80] Olson-Buchanan J. B. Boswell W. R. (2006). Blurring boundaries: correlates of integration and segmentation between work and nonwork. J. Vocat. Behav. 68, 432–445. doi: 10.1016/j.jvb.2005.10.006

[ref81] Palm E. Seubert C. Glaser J. (2020). Understanding employee motivation for work-to-nonwork integration behavior: a reasoned action approach. J. Bus. Psychol. 35, 683–696. doi: 10.1007/s10869-019-09648-5

[ref82] Park Y. Fritz C. Jex S. M. (2011). Relationships between work-home segmentation and psychological detachment from work: the role of communication technology use at home. J. Occup. Health Psychol. 16, 457–467. doi: 10.1037/a0023594, PMID: 21728434

[ref83] Perry S. J. Carlson D. S. Kacmar K. M. Wan M. Thompson M. J. (2022). Interruptions in remote work: a resource-based model of work and family stress. J. Bus. Psychol. doi: 10.1007/s10869-022-09842-y [E-pub ahead of print]., PMID: 36189432 PMC9510213

[ref84] Pfeffer I. Englert C. Mueller-Alcazar A. (2020). Perceived stress and trait self-control interact with the intention–behavior gap in physical activity behavior. Sport Exerc. Perform. Psychol. 9, 244–260. doi: 10.1037/spy0000189

[ref85] Piszczek M. M. Berg P. (2020). HR policy attribution: implications for work-family person-environment fit. Hum. Resour. Manag. Rev. 30:100701. doi: 10.1016/j.hrmr.2019.100701

[ref86] Podsakoff P. M. MacKenzie S. B. Lee J.-Y. Podsakoff N. P. (2003). Common method biases in behavioral research: a critical review of the literature and recommended remedies. J. Appl. Psychol. 88, 879–903. doi: 10.1037/0021-9010.88.5.879, PMID: 14516251

[ref87] Porter C. O. L. H. Outlaw R. Gale J. P. Cho T. S. (2019). The use of online panel data in management research: a review and recommendations. J. Manag. 45, 319–344. doi: 10.1177/0149206318811569

[ref88] Powell G. N. Greenhaus J. H. (2010). Sex, gender, and the work-to-family interface: exploring negative and positive interdependencies. AMJ 53, 513–534. doi: 10.5465/amj.2010.51468647

[ref89] R Core Team (2022). R: a language and environment for statistical computing. Available at: https://www.R-project.org/.

[ref90] Reif J. Spieß E. Pfaffinger K. (2021). Dealing with stress in a modern work environment: resources matter. Cham, Switzerland: Springer.

[ref91] Reinke K. Gerlach G. I. (2022). Linking availability expectations, bidirectional boundary management behavior and preferences, and employee well-being: an integrative study approach. J. Bus. Psychol. 37, 695–715. doi: 10.1007/s10869-021-09768-x

[ref92] Rexroth M. Feldmann E. Peters A. Sonntag K. (2016). Learning how to manage the boundaries between life domains: effects of a boundary management intervention on boundary management, recovery, and well-being. Z. Arb. Organ. 60, 117–129. doi: 10.1026/0932-4089/a000197

[ref93] Rofcanin Y. Anand S. (2020). Flexible work practices and work-family domain. Hum. Relat. 73, 1182–1185. doi: 10.1177/0018726720935778

[ref94] Rothbard N. P. Beetz A. M. Harari D. (2021). Balancing the scales: a configurational approach to work-life balance. Annu. Rev. Organ. Psychol. Organ. Behav. 8, 73–103. doi: 10.1146/annurev-orgpsych-012420-061833

[ref95] Rothbard N. P. Ollier-Malaterre A. (2016). “Boundary management” in The Oxford handbook of work and family. eds. Allen T. D. Eby L. T. (Oxford, England: Oxford University Press), 109–122.

[ref96] Rothbard N. P. Phillips K. W. Dumas T. L. (2005). Managing multiple roles: work-family policies and individuals’ desires for segmentation. Organ. Sci. 16, 243–258. doi: 10.1287/orsc.1050.0124

[ref97] Sheeran P. Webb T. L. (2016). The intention-behavior gap. Soc. Personal. Psychol. Compass 10, 503–518. doi: 10.1111/spc3.12265

[ref98] Shrout P. E. Lane S. P. (2012). “Psychometrics” in Handbook of research methods for studying daily life. eds. Mehl M. R. Conner T. S. (New York: Guilford), 302–320.

[ref99] Smith C. E. Wayne J. H. Matthews R. A. Lance C. E. Griggs T. L. Pattie M. W. (2022). Stability and change in levels of work–family conflict: a multi-study, longitudinal investigation. J. Occup. Organ. Psychol. 95, 1–35. doi: 10.1111/joop.12372

[ref100] Sonnentag S. Binnewies C. Mojza E. J. (2008). “Did you have a nice evening?” a day-level study on recovery experiences, sleep, and affect. J. Appl. Psychol. 93, 674–684. doi: 10.1037/0021-9010.93.3.674, PMID: 18457495

[ref101] Staines G. L. (1980). Spillover versus compensation: a review of the literature on the relationship between work and nonwork. Hum. Relat. 33, 111–129. doi: 10.1177/001872678003300203

[ref102] Stryker S. Burke P. J. (2000). The past, present, and future of an identity theory. Soc. Psychol. Q. 63, 284–297. doi: 10.2307/2695840

[ref1001] Tabachnick B. G. Fidell L. S. (2014). Using multivariate statistics. London, England: Pearson.

[ref103] ten Brummelhuis L. L. Bakker A. B. (2012). A resource perspective on the work–home interface: the work–home resources model. Am. Psychol. 67, 545–556. doi: 10.1037/a0027974, PMID: 22506688

[ref104] Thoits P. A. (1992). Identity structures and psychological well-being: gender and marital status comparisons. Soc. Psychol. Q. 55, 236–256. doi: 10.2307/2786794

[ref105] van Steenbergen E. F. Ybema J. F. Lapierre L. M. (2018). Boundary management in action: a diary study of students’ school-home conflict. Int. J. Stress. Manag. 25, 267–282. doi: 10.1037/str0000064

[ref106] Voydanoff P. (2004). The effects of work demands and resources on work-to-family conflict and facilitation. J. Marriage Fam. 66, 398–412. doi: 10.1111/j.1741-3737.2004.00028.x

[ref107] Voydanoff P. (2005). Consequences of boundary-spanning demands and resources for work-to-family conflict and perceived stress. J. Occup. Health Psychol. 10, 491–503. doi: 10.1037/1076-8998.10.4.491, PMID: 16248695

[ref108] Wayne J. H. Butts M. M. Casper W. J. Allen T. D. (2017). In search of balance: a conceptual and empirical integration of multiple meanings of work-family balance. Pers. Psychol. 70, 167–210. doi: 10.1111/peps.12132

[ref109] Wepfer A. G. Allen T. D. Brauchli R. Jenny G. J. Bauer G. F. (2018). Work-life boundaries and well-being: does work-to-life integration impair well-being through lack of recovery? J. Bus. Psychol. 33, 727–740. doi: 10.1007/s10869-017-9520-y

[ref110] Williams K. J. Alliger G. M. (1994). Role stressors, mood spillover, and perceptions of work-family conflict in employed parents. AMJ 37, 837–868. doi: 10.2307/256602

