# Supplementary material for: Effects of strain on boundary management: findings from a daily diary study and an experimental vignette study
Source: Front Psychol. 2023 Oct 24;14:1149969. doi: 10.3389/fpsyg.2023.1149969 (PMC10628038; doi:10.3389/fpsyg.2023.1149969)
Supplement: Supplementary file 1 [file Data_Sheet_1.docx]

Supplementary Material

Effects of Strain on Boundary Management: Findings from a Daily Diary Study and an Experimental Vignette Study

Nicolas Mueller*, Sophia Loeffelsend, Elke Vater and Regina Kempen

*** Correspondence:** Nicolas Mueller, nicolas.mueller@hs-aalen.de

**Table S1.** *Difference Tests Between Excluded and Included Participants [Study 1]*

| Effect | Excluded participants | Included participants | Difference test | | |
| --- | --- | --- | --- | --- | --- |
|  | *M* (*SD*) or *n* (%) | *M* (*SD*) or *n* (%) | *t* or χ^2^ | *df* | *p* |
| *t*-tests for comparing means |  |  |  |  |  |
| Work role involvement | 4.03 (1.31) | 3.96 (1.15) | 0.47 | 498 | .637 |
| Nonwork role involvement | 5.38 (1.21) | 5.33 (1.09) | 0.38 | 498 | .707 |
| Age | 43.27 (14.24) | 43.50 (13.00) | 0.14 | 498 | .886 |
| Working hours per week | 40.53 (5.24) | 40.79 (4.32) | 0.46 | 498 | .649 |
| Organizational tenure | 12.29 (11.20) | 12.29 (11.00) | < 0.01 | 498 | .998 |
| Work-related availability | 2.71 (1.06) | 2.59 (0.99) | 0.96 | 498 | .337 |
| Teleworking days per week | 1.41 (2.06) | 1.67 (2.08) | 0.97 | 498 | .333 |
| Impact of the COVID-19 pandemic | 2.67 (1.11) | 2.61 (1.04) | 0.40 | 498 | .690 |
|  |  |  |  |  |  |
| χ^2^-tests for comparing frequencies |  |  |  |  |  |
| Gender ^a^ | 36 (48.0) | 227 (53.4) | 0.55 | 1 | .459 |
| Living in a partnership ^b^ | 50 (66.7) | 308 (72.5) | 0.79 | 1 | .374 |
| Care for children ^b^ | 21 (28.0) | 101 (23.8) | 0.41 | 1 | .521 |
| Care for other people ^b^ | 9 (12.0) | 33 (7.8) | 0.99 | 1 | .321 |
| Self-employment ^b^ | 3 (4.0) | 33 (7.8) | 0.85 | 1 | .357 |
| Supervisory role ^b^ | 26 (34.7) | 131 (30.8) | 0.28 | 1 | .599 |
| Education level ^c^ | — | — | 3.18 | 3 | .365 |

*Note.* Excluded participants: *n*= 75; included participants: *n*= 425.

^a^ Coding: 0 = woman; 1 = man. ^b^ Coding: 0 = no; 1 = yes. ^c^ Coding: 1 = secondary modern school qualification, 2 = secondary school leaving certificate, 3 = high school diploma or a university of applied sciences entrance qualification, 4 = university degree.

**Table S2.** *Results of Multilevel Confirmatory Factor Analyses (MCFA) and Comparison of Model Fit [Study 1]*

| Model | Model specification | χ^2^ | *df* | *p* | CFI | TLI | RMSEA | SRMR_within_ | SRMR_between_ |
| --- | --- | --- | --- | --- | --- | --- | --- | --- | --- |
| MCFA results |  |  |  |  |  |  |  |  |  |
| 8-factor model (8) | All study variables separately | 1,090.49 | 139 | < .001 | 0.99 | 0.98 | 0.05 | 0.02 | 0.08 |
| 7-factor model (7a) | Work + nonwork strain | 14,336.41 | 144 | < .001 | 0.78 | 0.72 | 0.17 | 0.12 | 0.08 |
| 7-factor model (7b) | Work-to-nonwork + nonwork-to-work  segmentation preferences | 9,616.99 | 144 | < .001 | 0.85 | 0.82 | 0.14 | 0.10 | 0.08 |
| 7-factor model (7c) | Work-to-nonwork + nonwork-to-work  integration enactments | 5,807.22 | 144 | < .001 | 0.91 | 0.89 | 0.11 | 0.08 | 0.08 |
| 7-factor model (7d) | Work + nonwork role involvement | 1,419.38 | 140 | < .001 | 0.98 | 0.97 | 0.05 | 0.02 | 0.14 |
| 6-factor model (6) | Segmentation preferences + integration enactments | 14,451.91 | 148 | < .001 | 0.78 | 0.73 | 0.17 | 0.20 | 0.08 |
| 4-factor model (4) | (7a) + (7b) + (7c) + (7d) | 27,426.34 | 152 | < .001 | 0.58 | 0.50 | 0.24 | 0.16 | 0.14 |
| 3-factor model (3) | (4) + (segmentation preferences + integration enactments) | 35,438.04 | 154 | < .001 | 0.45 | 0.36 | 0.27 | 0.19 | 0.14 |
|  |  |  |  |  |  |  |  |  |  |
| Comparison of model fit | |  |  |  |  |  |  |  |  |
| (8) versus (7a) |  | 13,245.92 | 5 | < .001 |  |  |  |  |  |
| (8) versus (7b) |  | 8,526.50 | 5 | < .001 |  |  |  |  |  |
| (8) versus (7c) |  | 4,716.73 | 5 | < .001 |  |  |  |  |  |
| (8) versus (7d) |  | 328.89 | 1 | < .001 |  |  |  |  |  |
| (8) versus (6) |  | 1,3361.42 | 9 | < .001 |  |  |  |  |  |
| (8) versus (4) |  | 26,335.85 | 13 | < .001 |  |  |  |  |  |
| (8) versus (3) |  | 34,347.55 | 15 | < .001 |  |  |  |  |  |

*Note.* CFI = comparative fit index; TLI = Tucker-Lewis index; RMSEA = root-mean-square error of approximation; SRMR = standardized root-mean-square residual.

**Table S3.** *Results of Multilevel Modeling Analyses for Work-to-Nonwork Segmentation Preference Controlling for the Lagged Criterion [Study 1]*

| Effect |  |  | Model |  |  |
| --- | --- | --- | --- | --- | --- |
|  | Null | Control | Random intercept and fixed slope | Random intercept and random slope | Cross-level interaction |
| Intercept | 5.538^***^ (0.062) | 5.808^***^ (0.137) | 5.789^***^ (0.131) | 5.810^***^ (0.130) | 5.810^***^ (0.130) |
| Lagged w-to-n segmentation preference |  | –0.093^***^ (0.022) | –0.092^***^ (0.022) | –0.071^*^ (0.029) | –0.071^*^ (0.029) |
| Age |  | 0.011^*^ (0.005) | 0.014^**^ (0.005) | 0.012^*^ (0.005) | 0.012^*^ (0.005) |
| Education level ^a^ |  | –0.128 (0.065) | –0.120 (0.062) | –0.129^*^ (0.061) | –0.129^*^ (0.061) |
| Self-employment ^b^ |  | –0.445 (0.239) | –0.358 (0.229) | –0.420 (0.226) | –0.419 (0.226) |
| Availability |  | –0.126^*^ (0.063) | –0.074 (0.066) | –0.072 (0.065) | –0.073 (0.065) |
| Work role involvement |  |  | –0.010 (0.063) | –0.015 (0.062) | –0.008 (0.063) |
| Nonwork role involvement |  |  | 0.318^***^ (0.062) | 0.312^***^ (0.061) | 0.317^***^ (0.062) |
| Strain work |  |  | 0.004^***^ (0.001) | 0.005^**^ (0.002) | 0.005^**^ (0.002) |
| Strain nonwork |  |  | 0.000 (0.001) | 0.001 (0.002) | 0.001 (0.002) |
| Strain work × work role involv. |  |  |  |  | –0.002 (0.002) |
| Strain work × nonwork role involv. |  |  |  |  | –0.001 (0.002) |
| Strain nonwork × work role involv. |  |  |  |  | 0.003 (0.002) |
| Strain nonwork × nonwork role involv. |  |  |  |  | 0.002 (0.002) |
|  |  |  |  |  |  |
| Within-person variance | 0.844 | 0.837 | 0.833 | 0.641 | 0.641 |
| Intercept variance | 1.371 | 1.286 | 1.165 | 1.189 | 1.190 |
| Slope variance \| strain work |  |  |  | 0.0003 | 0.0003 |
| Slope variance \| strain nonwork |  |  |  | 0.0001 | 0.0001 |
| Slope variance \| lagged variable |  |  |  | 0.073 | 0.074 |
| Intercept-slope correlation \| strain work |  |  |  | –.32 | –.32 |
| Intercept-slope correlation \| strain nonwork |  |  |  | –.09 | –.11 |
| Intercept-slope correlation \| lagged variable |  |  |  | –.03 | –.03 |
|  |  |  |  |  |  |
| ICC | .619 |  |  |  |  |
| Deviance | 7,524.7 | 7,485.8 | 7,439.3 | 7,253.3 | 7,248.8 |
| Marginal *R*^2^ | — | .039 | .097 | .095 | .096 |
| Conditional *R*^2^ | .619 | .621 | .624 | .711 | .711 |

*Note.* Level-1 *N* = 2,476 and Level-2 *N* = 399. Unstandardized regression coefficients with standard errors in parentheses are reported. Maximum likelihood estimation of model parameters was used. W-to-n = work-to-nonwork; involv. = involvement; ICC = intraclass correlation.

^a^ Treated as a numeric variable from 0 = no graduation to 4 = university degree. ^b^ Coding: 0 = no; 1 = yes.

^*^*p* < .05. ^**^*p* < .01. ^***^*p* < .001.

**Table S4.** *Results of Multilevel Modeling Analyses for Nonwork-to-Work Segmentation Preference Controlling for the Lagged Criterion [Study 1]*

| Effect |  |  | Model |  |  |
| --- | --- | --- | --- | --- | --- |
|  | Null | Control | Random intercept and fixed slope | Random intercept and random slope | Cross-level interaction |
| Intercept | 4.564^***^ (0.068) | 4.833^***^ (0.126) | 4.831^***^ (0.126) | 4.789^***^ (0.125) | 4.787^***^ (0.125) |
| Lagged n-to-w segmentation preference |  | 0.059^**^ (0.021) | 0.057^**^ (0.021) | 0.026 (0.025) | 0.026 (0.024) |
| Age |  | 0.015^*^ (0.006) | 0.014^*^ (0.006) | 0.013^*^ (0.006) | 0.013^*^ (0.006) |
| Partnership ^a^ |  | –0.378^*^ (0.148) | –0.376^*^ (0.149) | –0.323^*^ (0.148) | –0.320^*^ (0.148) |
| Organizational tenure |  | 0.009 (0.007) | 0.010 (0.007) | 0.010 (0.007) | 0.010 (0.007) |
| Work role involvement |  |  | 0.121 (0.066) | 0.116 (0.065) | 0.124 (0.066) |
| Nonwork role involvement |  |  | 0.014 (0.071) | 0.022 (0.070) | 0.015 (0.071) |
| Strain work |  |  | 0.001 (0.001) | 0.003 (0.002) | 0.003^*^ (0.002) |
| Strain nonwork |  |  | 0.002 (0.001) | 0.003 (0.002) | 0.003 (0.002) |
| Strain work × work role involv. |  |  |  |  | –0.001 (0.002) |
| Strain work × nonwork role involv. |  |  |  |  | –0.003 (0.002) |
| Strain nonwork × work role involv. |  |  |  |  | –0.002 (0.003) |
| Strain nonwork × nonwork role involv. |  |  |  |  | 0.002 (0.003) |
|  |  |  |  |  |  |
| Within-person variance | 1.012 | 1.007 | 1.005 | 0.790 | 0.790 |
| Intercept variance | 1.629 | 1.542 | 1.526 | 1.553 | 1.553 |
| Slope variance \| strain work |  |  |  | 0.0002 | 0.0001 |
| Slope variance \| strain nonwork |  |  |  | 0.0007 | 0.0007 |
| Slope variance \| lagged variable |  |  |  | 0.035 | 0.034 |
| Intercept-slope correlation \| strain work |  |  |  | –.01 | –.02 |
| Intercept-slope correlation \| strain nonwork |  |  |  | –.29 | –.29 |
| Intercept-slope correlation \| lagged variable |  |  |  | –.12 | –.13 |
|  |  |  |  |  |  |
| ICC | .617 |  |  |  |  |
| Deviance | 7,970.1 | 7,941.6 | 7,934.4 | 7,768.0 | 7,762.8 |
| Marginal *R*^2^ | — | .037 | .044 | .037 | .040 |
| Conditional *R*^2^ | .617 | .619 | .620 | .701 | .702 |

*Note.* Level-1 *N* = 2,476 and Level-2 *N* = 399. Unstandardized regression coefficients with standard errors in parentheses are reported. Maximum likelihood estimation of model parameters was used. N-to-w = nonwork-to-work; involv. = involvement; ICC = intraclass correlation.

^a^ Coding: 0 = no; 1 = yes.

^*^*p* < .05. ^**^*p* < .01. ^***^*p* < .001.

**Table S5.** *Results of Multilevel Modeling Analyses for Work-to-Nonwork Integration Enactment Controlling for the Lagged Criterion [Study 1]*

| Effect |  |  | Model |  |  |
| --- | --- | --- | --- | --- | --- |
|  | Null | Control | Random intercept and fixed slope | Random intercept and random slope | Cross-level interaction |
| Intercept | 2.846^***^ (0.065) | 2.746^***^ (0.132) | 2.763^***^ (0.126) | 2.776^***^ (0.126) | 2.776^***^ (0.126) |
| Lagged w-to-n integration enactment |  | –0.032 (0.022) | –0.032 (0.021) | –0.042 (0.023) | –0.042 (0.023) |
| Age |  | –0.014^*^ (0.006) | –0.019^***^ (0.005) | –0.018^***^ (0.005) | –0.018^***^ (0.005) |
| Working hours |  | 0.032^*^ (0.014) | 0.027^*^ (0.013) | 0.026 (0.013) | 0.026^*^ (0.013) |
| Education level ^a^ |  | 0.062 (0.063) | 0.056 (0.060) | 0.047 (0.060) | 0.047 (0.060) |
| Organizational tenure |  | –0.006 (0.007) | –0.002 (0.006) | –0.003 (0.006) | –0.003 (0.006) |
| Availability |  | 0.374^***^ (0.061) | 0.290^***^ (0.064) | 0.293^***^ (0.063) | 0.294^***^ (0.063) |
| COVID-19 impact |  | 0.168^**^ (0.058) | 0.194^***^ (0.056) | 0.192^***^ (0.055) | 0.192^***^ (0.055) |
| Work role involvement |  |  | 0.095 (0.061) | 0.085 (0.060) | 0.093 (0.061) |
| Nonwork role involvement |  |  | –0.240^***^ (0.060) | –0.245^***^ (0.059) | –0.241^***^ (0.060) |
| Strain work |  |  | 0.014^***^ (0.001) | 0.012^***^ (0.002) | 0.012^***^ (0.002) |
| Strain nonwork |  |  | 0.006^***^ (0.002) | 0.007^***^ (0.002) | 0.007^**^ (0.002) |
| Strain work × work role involv. |  |  |  |  | 0.003 (0.002) |
| Strain work × nonwork role involv. |  |  |  |  | 0.001 (0.002) |
| Strain nonwork × work role involv. |  |  |  |  | –0.001 (0.002) |
| Strain nonwork × nonwork role involv. |  |  |  |  | 0.000 (0.002) |
|  |  |  |  |  |  |
| Within-person variance | 1.230 | 1.229 | 1.163 | 0.984 | 0.985 |
| Intercept variance | 1.403 | 1.074 | 0.980 | 1.008 | 1.007 |
| Slope variance \| strain work |  |  |  | 0.0004 | 0.0004 |
| Slope variance \| strain nonwork |  |  |  | 0.0003 | 0.0003 |
| Slope variance \| lagged variable |  |  |  | 0.016 | 0.015 |
| Intercept-slope correlation \| strain work |  |  |  | .20 | .21 |
| Intercept-slope correlation \| strain nonwork |  |  |  | –.07 | –.07 |
| Intercept-slope correlation \| lagged variable |  |  |  | .28 | .30 |
|  |  |  |  |  |  |
| ICC | .533 |  |  |  |  |
| Deviance | 8,336.2 | 8,245.0 | 8,097.8 | 8,005.7 | 8,003.5 |
| Marginal *R*^2^ | — | .128 | .189 | .184 | .187 |
| Conditional *R*^2^ | .533 | .535 | .560 | .627 | .628 |

*Note.* Level-1 *N* = 2,476 and Level-2 *N* = 399. Unstandardized regression coefficients with standard errors in parentheses are reported. Maximum likelihood estimation of model parameters was used. W-to-n = work-to-nonwork; involv. = involvement; ICC = intraclass correlation.

^a^ Treated as a numeric variable from 0 = no graduation to 4 = university degree.

^*^*p* < .05. ^**^*p* < .01. ^***^*p* < .001.

**Table S6.** *Results of Multilevel Modeling Analyses for Nonwork-to-Work Integration Enactment Controlling for the Lagged Criterion [Study 1]*

| Effect |  |  | Model |  |  |
| --- | --- | --- | --- | --- | --- |
|  | Null | Control | Random intercept and fixed slope | Random intercept and random slope | Cross-level interaction |
| Intercept | 3.004^***^ (0.062) | 2.945^***^ (0.134) | 2.956^***^ (0.132) | 2.962^***^ (0.132) | 2.960^***^ (0.132) |
| Lagged n-to-w integration enactment |  | –0.008 (0.021) | 0.001 (0.021) | –0.011 (0.023) | –0.009 (0.023) |
| Age |  | –0.012^*^ (0.006) | –0.015^**^ (0.006) | –0.013^*^ (0.006) | –0.013^*^ (0.006) |
| Education level ^a^ |  | 0.038 (0.064) | 0.031 (0.063) | 0.030 (0.063) | 0.031 (0.063) |
| Organizational tenure |  | –0.013^*^ (0.007) | –0.011 (0.007) | –0.012 (0.007) | –0.012 (0.007) |
| Availability |  | 0.170^**^ (0.061) | 0.136^*^ (0.066) | 0.132^*^ (0.066) | 0.131^*^ (0.066) |
| COVID-19 impact |  | 0.177^**^ (0.058) | 0.195^***^ (0.058) | 0.194^***^ (0.058) | 0.193^**^ (0.058) |
| Work role involvement |  |  | 0.017 (0.064) | 0.017 (0.064) | 0.018 (0.064) |
| Nonwork role involvement |  |  | –0.123 (0.063) | –0.104 (0.063) | –0.112 (0.063) |
| Strain work |  |  | –0.001 (0.001) | –0.001 (0.002) | –0.001 (0.002) |
| Strain nonwork |  |  | 0.022^***^ (0.002) | 0.025^***^ (0.002) | 0.025^***^ (0.002) |
| Strain work × work role involv. |  |  |  |  | 0.001 (0.002) |
| Strain work × nonwork role involv. |  |  |  |  | 0.002 (0.002) |
| Strain nonwork × work role involv. |  |  |  |  | 0.000 (0.002) |
| Strain nonwork × nonwork role involv. |  |  |  |  | 0.004 (0.003) |
|  |  |  |  |  |  |
| Within-person variance | 1.262 | 1.261 | 1.152 | 0.954 | 0.954 |
| Intercept variance | 1.282 | 1.113 | 1.100 | 1.141 | 1.139 |
| Slope variance \| strain work |  |  |  | 0.0002 | 0.0002 |
| Slope variance \| strain nonwork |  |  |  | 0.0006 | 0.0006 |
| Slope variance \| lagged variable |  |  |  | 0.031 | 0.030 |
| Intercept-slope correlation \| strain work |  |  |  | –.02 | –.03 |
| Intercept-slope correlation \| strain nonwork |  |  |  | –.20 | –.23 |
| Intercept-slope correlation \| lagged variable |  |  |  | –.04 | –.03 |
|  |  |  |  |  |  |
| ICC | .504 |  |  |  |  |
| Deviance | 8,360.3 | 8,311.7 | 8,115.0 | 8,015.5 | 8,010.3 |
| Marginal *R*^2^ | — | .070 | .116 | .119 | .121 |
| Conditional *R*^2^ | .504 | .506 | .548 | .632 | .632 |

*Note.* Level-1 *N* = 2,476 and Level-2 *N* = 399. Unstandardized regression coefficients with standard errors in parentheses are reported. Maximum likelihood estimation of model parameters was used. N-to-w = nonwork-to-work; involv. = involvement; ICC = intraclass correlation.

^a^ Treated as a numeric variable from 0 = no graduation to 4 = university degree.

^*^*p* < .05. ^**^*p* < .01. ^***^*p* < .001.

**Table S7.** *Vignettes Used in Study 2*

| Vignette | Strain in  work life | Strain in  nonwork life | *n* | Description |
| --- | --- | --- | --- | --- |
| 1 | + | + | 58 | Please think of a situation where you would perceive **much** strain in your work life. For example, you face **high** time and performance pressures, **many** work tasks to handle, and **some** conflicts with colleagues. Furthermore, imagine that you would perceive **much** strain in your nonwork life. For example, you face **many** private meetings and responsibilities, **many** nonwork tasks to handle, and **some** conflicts with family members. |
| 2 | + | – | 42 | Please think of a situation where you would perceive **much** strain in your work life. For example, you face **high** time and performance pressures, **many** work tasks to handle, and **some** conflicts with colleagues. Furthermore, imagine that you would perceive **less** strain in your nonwork life. For example, you face **few** private meetings and responsibilities, **few** nonwork tasks to handle, and **no** conflicts with family members. |
| 3 | – | + | 52 | Please think of a situation where you would perceive **less** strain in your work life. For example, you face **low** time and performance pressures, **few** work tasks to handle, and **no** conflicts with colleagues. Furthermore, imagine that you would perceive **much** strain in your nonwork life. For example, you face **many** private meetings and responsibilities, **many** nonwork tasks to handle, and **some** conflicts with family members. |
| 4 | – | – | 29 | Please think of a situation where you would perceive **less** strain in your work life. For example, you face **low** time and performance pressures, **few** work tasks to handle, and **no** conflicts with colleagues. Furthermore, imagine that you would perceive **less** strain in your nonwork life. For example, you face **few** private meetings and responsibilities, **few** nonwork tasks to handle, and **no** conflicts with family members. |

*Note.* Differences in the vignette descriptions are in bold. Plus sign (+) = much strain; minus sign (–) = less strain.

**Table S8.** *Data Transparency Regarding the Variables Collected in the Experimental Vignette Study but not Used in Study 2*

| Variables in the complete dataset | Used in Study 2 | | Collected but not used  in Study 2 |
| --- | --- | --- | --- |
|  | Hypothesis test | Manipulation and randomization check |  |
| Actual work-to-nonwork segmentation preference |  | ✓ |  |
| Actual nonwork-to-work segmentation preference |  | ✓ |  |
| Hypothetical work-to-nonwork segmentation preference | ✓ |  |  |
| Hypothetical nonwork-to-work segmentation preference | ✓ |  |  |
| Hypothetical strain in work life |  | ✓ |  |
| Hypothetical strain in nonwork life |  | ✓ |  |
| Hypothetical coping in work life |  |  | ✓ |
| Hypothetical coping in nonwork life |  |  | ✓ |
| Work role involvement |  |  | ✓ |
| Nonwork role involvement |  |  | ✓ |
|  |  |  |  |
| Sociodemographic variables |  |  |  |
| Gender | ✓ |  |  |
| Age | ✓ |  |  |
| Living in a partnership | ✓ |  |  |
| Care for children | ✓ |  |  |
| Care for parents | ✓ |  |  |
| Education level | ✓ |  |  |
| Employment | ✓ |  |  |
| Self-employment | ✓ |  |  |
| Supervisory role | ✓ |  |  |
| Organizational tenure | ✓ |  |  |
| Working hours | ✓ |  |  |
| Sector (i.e., professional industry) | ✓ |  |  |

*Note.* Variables collected but not used in Study 2 are planned to be used in another manuscript that addresses another research question. **Table S9.** *Results of Confirmatory Factor Analyses (CFA) and Comparison of Model Fit [Study 2]*

| Model | Model specification | χ^2^ | *df* | *p* | CFI | TLI | RMSEA | SRMR |
| --- | --- | --- | --- | --- | --- | --- | --- | --- |
| CFA results |  |  |  |  |  |  |  |  |
| 6-factor model (6) | All study variables separately | 300.00 | 194 | < .001 | 0.97 | 0.97 | 0.06 | 0.04 |
| 5-factor model (5a) | Work + nonwork strain | 845.85 | 199 | < .001 | 0.83 | 0.80 | 0.13 | 0.11 |
| 5-factor model (5b) | Hypothetical work-to-nonwork segmentation preference + work strain | 839.04 | 199 | < .001 | 0.83 | 0.80 | 0.13 | 0.10 |
| 5-factor model (5c) | Hypothetical nonwork-to-work segmentation preference + nonwork strain | 794.41 | 199 | < .001 | 0.84 | 0.81 | 0.13 | 0.09 |
| 4-factor model (4a) | Work-to-nonwork + nonwork-to-work  segmentation preferences | 1,254.37 | 203 | < .001 | 0.72 | 0.68 | 0.17 | 0.17 |
| 4-factor model (4b) | Actual + hypothetical  segmentation preferences | 1,056.99 | 203 | < .001 | 0.78 | 0.74 | 0.15 | 0.15 |
| 3-factor model (3) | (4a) + (4b) | 1,798.78 | 206 | < .001 | 0.57 | 0.52 | 0.21 | 0.18 |
| 2-factor model (2) | (5a) + (3) | 2,312.93 | 208 | < .001 | 0.43 | 0.37 | 0.24 | 0.20 |
|  |  |  |  |  |  |  |  |  |
| Comparison of model fit | |  |  |  |  |  |  |  |
| (6) versus (5a) |  | 545.85 | 5 | < .001 |  |  |  |  |
| (6) versus (5b) |  | 539.04 | 5 | < .001 |  |  |  |  |
| (6) versus (5c) |  | 494.41 | 5 | < .001 |  |  |  |  |
| (6) versus (4a) |  | 954.37 | 9 | < .001 |  |  |  |  |
| (6) versus (4b) |  | 756.99 | 9 | < .001 |  |  |  |  |
| (6) versus (3) |  | 1,498.78 | 12 | < .001 |  |  |  |  |
| (6) versus (2) |  | 2,012.94 | 14 | < .001 |  |  |  |  |

*Note.* CFI = comparative fit index; TLI = Tucker-Lewis index; RMSEA = root-mean-square error of approximation; SRMR = standardized root-mean-square residual.

**Table S10.** *Results of Two-Way Analyses of Variance With Transformed Segmentation Preference Scores [Study 2]*

| Effect | Work-to-nonwork segmentation preference | | | | Nonwork-to-work segmentation preference | | | |
| --- | --- | --- | --- | --- | --- | --- | --- | --- |
|  | *F* or *t* | *df* | *p* | η^2^_p_ | *F* or *t* | *df* | *p* | η^2^_p_ |
| Work strain | 34.51 | 1, 177 | < .001 | .16 | 17.48 | 1, 177 | < .001 | .09 |
| Nonwork strain | 1.15 | 1, 177 | .285 | .01 | 31.30 | 1, 177 | < .001 | .15 |
| Work strain × nonwork strain | 0.26 | 1, 177 | .613 | < .01 | 5.42 | 1, 177 | .021 | .03 |
|  |  |  |  |  |  |  |  |  |
| Simple main effects for work strain |  |  |  |  |  |  |  |  |
| Less nonwork strain | 3.42 | 177 | < .001 |  | 4.15 | 177 | < .001 |  |
| Much nonwork strain | 5.14 | 177 | < .001 |  | 1.49 | 177 | .137 |  |
| Simple main effects for nonwork strain |  |  |  |  |  |  |  |  |
| Less work strain | 0.38 | 177 | .708 |  | 5.26 | 177 | < .001 |  |
| Much work strain | 1.20 | 177 | .232 |  | 2.48 | 177 | .014 |  |

*Note.* *N* = 181. Segmentation preference sores were transformed by reflecting them and applying a square root transformation [i.e., score_transformed_ = (scale maximum + 1 – score_untransformed_)^1/2^].
